# Adjuvant Chemotherapy in Lymph Node‐Negative, T1 Triple‐Negative Breast Cancer

**DOI:** 10.1002/cam4.71347

**Published:** 2025-11-04

**Authors:** Jesus D. Anampa, Alvaro Alvarez Soto, Rachel B. Jimenez, Samilia Obeng‐Gyasi, Xiaonan Xue

**Affiliations:** ^1^ Department of Medical Oncology, Montefiore Einstein Comprehensive Cancer Center New York New York USA; ^2^ Department of Medicine, Hematology/Oncology, Carole and Ray Neag Comprehensive Cancer Center, UCONN Health Farmington Connecticut USA; ^3^ Department of Radiation Oncology, Massachusetts General Hospital Boston Massachusetts USA; ^4^ Department of Surgery, Division of Surgical Oncology The Ohio State University Columbus Ohio USA; ^5^ Department of Epidemiology and Population Health Albert Einstein College of Medicine New York New York USA

## Abstract

**Background:**

The role of adjuvant chemotherapy in small, node‐negative triple‐negative breast cancer (TNBC) has not been formally assessed in clinical trials. We aimed to evaluate the benefit of adjuvant chemotherapy in this population.

**Methods:**

This is a retrospective study using the National Cancer Database (NCDB). We included women with pathologic T1N0M0 (pT1N0M0) TNBC diagnosed between 2010 and 2019. Kaplan–Meier methods and Cox proportional models were used to compare overall survival (OS) between chemotherapy and no chemotherapy cohorts.

**Results:**

In patients with pT1N0M0 TNBC, adjuvant chemotherapy improved OS (HR, 0.51; 95% CI, 0.47–0.55). The benefit of adjuvant chemotherapy was evidenced across all tumor sizes; T1a HR 0.51 (95% CI 0.34–0.75), T1b HR 0.55 (95% CI 0.47–0.64), and T1c HR 0.48 (95% CI 0.43–0.53). Despite similar HRs associated with chemotherapy across tumor sizes, T1c had the worst OS without chemotherapy, thus the most gain in 5‐year OS: 5‐year OS rates for chemotherapy vs. no chemotherapy for T1a, T1b and T1c tumors were 98% vs. 93%, 96% vs. 89%, and 93% vs. 80%, respectively. Although there were no ethnoracial differences in chemotherapy receipt, Non‐Hispanic Black women (HR 0.65, 95% CI 0.54–0.76) derived less benefit compared to Non‐Hispanic White women (HR 0.49, 95% CI 0.44–0.53).

**Conclusions:**

Adjuvant chemotherapy improves survival in patients with pT1N0M0 TNBC. The magnitude of the benefit is greatest for T1c tumors than for T1b and T1a tumors. Future studies are required to identify biomarkers to select patients who derive the most chemotherapy benefit.

**Abbreviations**
BCS: breast‐conserving surgery
CCI: Charlson‐Deyo Comorbidity Index
Chemo: chemotherapy
CI: confidence interval
CoC: commission of cancer
ER: estrogen receptor
HER2: human epidermal growth factor receptor 2
HR: hazard ratio
K: thousand dollars
NCCN: National Comprehensive Cancer Network
NCDB: National Comprehensive Database
NH: non‐Hispanic
OR: odds ratio
OS: overall survival
PR: progesterone receptor
RR: relative risk
TNBC: triple‐negative breast cancer

## 1 Introduction

Breast cancer is the most common malignancy and the leading cause of cancer‐related death among women worldwide [1]. Triple‐negative breast cancer (TNBC) lacks estrogen‐receptor (ER) and progesterone‐receptor (PR) expression and human epidermal growth factor receptor 2 (HER2) amplification [2, 3] and accounts for 10%–20% of all breast cancers [4]. TNBC is associated with more advanced stages upon presentation and higher recurrence and mortality rates than other breast cancer subtypes [5, 6, 7, 8, 9]. Despite recent progress, 25%–30% of patients with early‐stage TNBC develop metastasis within 3 to 5 years of diagnosis [10]. In patients with TNBC who proceed with surgery upfront, adjuvant chemotherapy remains a cornerstone in the treatment [11, 12], demonstrating significant improvement in disease‐free survival and overall survival (OS) [13, 14].

Mammography screening has increased the detection of early breast cancers, identifying even TNBC at early stages of development [15]. The role of adjuvant chemotherapy in small, node‐negative TNBC has not been formally evaluated in prospective randomized clinical trials. Some retrospective studies suggest that patients with small, node‐negative TNBC achieve excellent long‐term outcomes regardless of adjuvant chemotherapy administration [16, 17, 18, 19, 20]. Conversely, other studies have reported that adjuvant chemotherapy is associated with improved breast cancer outcomes even in this population [21, 22].

Practice guidelines for the management of node‐negative, early breast cancer, such as the National Comprehensive Cancer Network (NCCN) and the St. Gallen International Expert Consensus on the Primary Therapy of Early Breast Cancer, recommend adjuvant chemotherapy for patients with TNBC and tumor size 11–20 mm (T1c). Chemotherapy is suggested for patients with tumors 6–10 mm (T1b). For tumors ≤ 5 mm (T1a), adjuvant chemotherapy is not routinely recommended unless the patient has high‐risk features [23, 24]. However, high‐quality data informing these recommendations is scarce. Therefore, we assessed the benefit of adjuvant chemotherapy in a nationwide dataset of patients with small size (T1), node‐negative TNBC (pT1N0M0). Moreover, we explored the variables associated with chemotherapy administration in these patients.

## 2 Methods

### 2.1 Data Collection

We conducted a retrospective cohort study. De‐identified data were extracted from the National Cancer Database (NCDB, participant user file 2020) [25, 26]. The study inclusion criteria were (1) female patients with breast cancer diagnosed from 2010 to 2019, (2) age ≥ 18 years, (3) tumor size ≤ 2 cm, (4) pathological evaluation of tumor size and nodal status, (5) no regional nodal metastasis (N0), (6) no distant metastasis (M0), (7) ER, PR, and HER2 negative, (8) underwent surgical removal of breast tumor, and, (9) no receipt of preoperative radiation or systemic therapy. Patients were excluded if follow‐up < 1 month, prior malignancies, bilateral breast cancer, positive surgical margins, or unknown tumor grade. The study was approved by the Albert Einstein Institutional Review Board and adhered to the Strengthening the Reporting of Observational Studies in Epidemiology (STROBE) reporting guidelines [27].

### 2.2 Variables

We obtained data on demographic variables such as diagnosis year, age, and race; this last variable was collected in combination with ethnicity, leading to 4 racial groups: non‐Hispanic White (NH White), non‐Hispanic Black (NH Black), Hispanics from all races, and non‐Hispanic Asian/Pacific islander and non‐Hispanic American Indian/Alaska Native (NH other). As the NCDB does not collect genetic ancestry data, ethnoracial categories in this study are a sociopolitical construct and not representative of genetic ancestry [28]. Sociodemographic variables collected included rurality, insurance, county median household income, county high school education, and facility type. Clinicopathological variables collected included the Charlson‐Deyo Comorbidity Index (CCI), tumor size, histology, and tumor grade. Treatment variables collected included surgery, chemotherapy, and radiotherapy.

### 2.3 Statistical Analysis

The primary endpoint of our study was OS. Based on chemotherapy receipt, we divided eligible patients into two groups: those who received chemotherapy versus those who received no chemotherapy. We used the Pearson's *X*‐square test to assess the association between categorical variables and the student *t*‐test for continuous variables. Logistic regression analyses were used to identify the factors associated with chemotherapy administration.

OS was defined as the time in months from diagnosis to death from any cause or last follow‐up for censored patients [25]. We estimated OS for patients with and without chemotherapy using Kaplan–Meier methods and compared their survival rates using the log‐rank test. We performed a multivariable Cox regression model to compare OS for chemotherapy and no chemotherapy groups, adjusting for clinicopathological, sociodemographic, and treatment variables. Schoenfeld residual plots were used to examine the proportional hazard assumption, and no obvious violation was identified. Hazard ratios (HRs) and 95% confidence intervals (CIs) were estimated from the model. To further control for potential confounding due to the observed differences among patients receiving chemotherapy vs. not, we adopted a stabilized inverse propensity weighted analysis based on propensity score [29, 30, 31] as a sensitivity analysis. The propensity score was calculated based on the logistic regression on chemotherapy versus not discussed above.

Stratified analyses were performed to examine the differences in OS for chemotherapy and no chemotherapy cohorts by age, race, histology, tumor grade, tumor size, radiation, and type of surgery. The interaction between chemotherapy and other prognostic factors was assessed by including a cross‐product term in the multivariable‐adjusted models. The likelihood ratio test was used to assess the interaction effects. Next, we conducted similar statistical methods to determine differences in OS for multiagent chemotherapy versus no chemotherapy.

Two‐sided *p* values and 95% CIs are reported. An *α* equal to 0.05 was used for all hypothesis testing. Statistical analyses were performed in R (Version 1.4.1106).

## 3 Results

Our study included 36,396 patients with pT1N0M0 TNBC (chemotherapy group, *n* = 25,462; no chemotherapy group, *n* = 10,934) (Figure S1). Most patients had T1c tumors (61%), followed by T1b (29%) and T1a (11%) tumors. Chemotherapy was administered in 80%, 65%, and 23% of patients with T1c, T1b, and T1a tumors, respectively. Baseline characteristics are summarized in Table 1. The median age was 62 years; most patients were NH White (71%), followed by NH Black (18%), NH other race (7%), and Hispanic (5%). Ductal histology composed 89% of all tumors, and most tumors were poorly differentiated (73%). Most patients had no major comorbidities (CCI = 0; 82%) and received care in a comprehensive cancer program (40%). Breast‐conserving surgery (BCS) [74%] was the most common surgical procedure. Adjuvant radiation was used in 91% and 3% of patients who underwent BCS and mastectomy, respectively. Regarding sociodemographic variables, most patients had private insurance (52%), high household income (> 74 K, 33%) and lived in a metropolitan county (84%).

**TABLE 1:** Baseline characteristics of patients with pT1N0M0 triple‐negative breast cancer.

Characteristics	Overall	Chemo	No Chemo	*p*
	(*n* = 36,396)	(*n* = 25,462)	(*n* = 10,934)	
Age, y, median (IQR)	62 (53–70)	59 (51–66)	69 (61–76)	
Age, y, no. (%)				< 0.001
50–70	22,216 (61%)	17,072 (67%)	5144 (47%)	
< 50	5876 (16%)	5123 (20%)	753 (7%)	
> 70	8304 (23%)	3267 (13%)	5037 (46%)	
Year of diagnosis				0.046
2010–2012	11,076 (30%)	7697 (30%)	3379 (31%)	
2013–2015	11,106 (31%)	7869 (31%)	3237 (30%)	
2016–2019	14,214 (39%)	9896 (39%)	4318 (39%)	
Race/ethnicity				< 0.001
NH White	25,754 (71%)	17,813 (70%)	7941 (73%)	
NH Black	6546 (18%)	4734 (19%)	1812 (17%)	
Hispanic	1729 (5%)	1275 (5%)	454 (4%)	
NH Other	2367 (7%)	1640 (6%)	727 (7%)	
Histology				< 0.001
Ductal	32,347 (89%)	23,057 (91%)	9290 (85%)	
Lobular	281 (1%)	136 (1%)	145 (1%)	
Ductolobular	282 (1%)	182 (1%)	100 (1%)	
Other	3486 (10%)	2087 (8%)	1399 (13%)	
Tumor Grade				< 0.001
Well differentiated	1256 (3%)	432 (2%)	824 (8%)	
Moderately differentiated	8406 (23%)	4727 (19%)	3679 (34%)	
Poorly differentiated/undifferentiated	26,734 (73%)	20,303 (80%)	6431 (59%)	
T1 stage				< 0.001
T1a	3845 (11%)	887 (3%)	2958 (27%)	
T1b	10,449 (29%)	6827 (27%)	3622 (33%)	
T1c	22,102 (61%)	17,748 (70%)	4354 (40%)	
Type of facility				< 0.001
Community cancer program	2616 (7%)	1777 (7%)	839 (8%)	
Comprehensive cancer program	14,500 (40%)	9891 (39%)	4609 (42%)	
Academic program	10,535 (29%)	7535 (30%)	3000 (27%)	
Integrated network cancer program	7406 (20%)	5061 (20%)	2345 (21%)	
Unknown	1339 (4%)	1198 (5%)	141 (1%)	
Rurality				0.113
Metro	30,693 (84%)	21,420 (84%)	9273 (85%)	
Urban–rural	4912 (13%)	3466 (14%)	1446 (13%)	
Unknown	791 (2%)	576 (2%)	215 (2%)	
Insurance				< 0.001
Medicaid/medicare	16,243 (45%)	9292 (36%)	6951 (64%)	
Private	18,932 (52%)	15,262 (60%)	3670 (34%)	
Uninsured	481 (1%)	358 (1%)	123 (1%)	
Other	740 (2%)	550 (2%)	190 (2%)	
Income				< 0.001
< 46k	5179 (14%)	3520 (14%)	1659 (15%)	
46k–57k	6589 (18%)	4529 (18%)	2060 (19%)	
58k–74k	7461 (20%)	5180 (20%)	2281 (21%)	
> 74k	12,181 (33%)	8725 (34%)	3456 (32%)	
Unknown	4986 (14%)	3508 (14%)	1478 (14%)	
No high‐school education				0.130
15.3%	6104 (17%)	4240 (17%)	1864 (17%)	
9.1%–15.2%	8718 (24%)	6056 (24%)	2662 (24%)	
5%–9%	9224 (25%)	6423 (25%)	2801 (26%)	
< 5%	7439 (20%)	5289 (21%)	2150 (20%)	
Unknown	4911 (13%)	3454 (14%)	1457 (13%)	
Comorbidities (CCI)				< 0.001
CCI = 0	29,725 (82%)	21,148 (83%)	8577 (78%)	
CCI = 1	5130 (14%)	3437 (13%)	1693 (15%)	
CCI = 2	1060 (3%)	626 (2%)	434 (4%)	
CCI ≥ 3	481 (1%)	251 (1%)	230 (2%)	
Surgery type				0.081
BCS	27,112 (74%)	18,900 (74%)	8212 (75%)	
Mastectomy	9284 (26%)	6562 (26%)	2722 (25%)	
Radiation in BCS				< 0.001
No	2353 (9%)	960 (5%)	1393 (17%)	
Yes	24,759 (91%)	17,940 (95%)	6819 (83%)	
Radiation in Mastectomy				< 0.001
No	9025 (97%)	6354 (97%)	2671 (98%)	
Yes	259 (3%)	208 (3%)	51 (2%)	

Abbreviations: BCS, breast‐conserving surgery; CCI, Charlson‐Deyo Comorbidity Index; IQR, interquartile range; k, thousand dollars; NH, non‐hispanic; No., number; TNBC, triple‐negative breast cancer; y, years.

Over the study period, there was no difference in the use of adjuvant chemotherapy from 2010 to 2019 (69% vs. 70%) (Figure S2). Furthermore, the use of adjuvant chemotherapy decreased (25% vs. 20%), increased (59% vs. 68%), and remained stable (80% vs. 81%) in patients with T1a, T1b, and T1c tumors during the study period, respectively (Figure S3 and Table S1).

### 3.1 Factors Associated With the Administration of Chemotherapy

Young patients (age < 50 years) had higher odds of receiving adjuvant chemotherapy (OR, 1.69; 95% CI, 1.52–1.88); conversely, older aged adults (age > 70 years) had lower odds of receiving adjuvant chemotherapy (OR, 0.19; 95% CI, 0.18–0.21). Compared to NH White women, NH Black and Hispanic women had similar odds of receiving adjuvant chemotherapy; however, NH other race had lower chances of chemotherapy administration (OR, 0.87; 95% CI, 0.78–0.97). Patients with T1b (OR, 9.12; 95% CI, 8.30–10.02) and T1c (OR, 22.25; 95% CI, 20.28–24.43) tumors had higher odds of receiving chemotherapy as compared to those with T1a tumors. Histology other than ductal (OR, 0.63; 95% CI, 0.58–0.68) was associated with decreased odds of chemotherapy administration, whereas high tumor grade (OR, 4.85; 95% CI, 4.20–5.60) was associated with higher odds of chemotherapy administration (Table 2).

**TABLE 2:** Factors associated with the use of chemotherapy in patients with pT1N0M0 triple‐negative breast cancer.

Characteristics	OR	95% CI	*p*
Age (years)			
50–70	Reference		
Age < 50	1.69	1.52–1.88	< 0.001
Age > 70	0.19	0.18–0.21	< 0.001
Race/ethnicity			
NH White	Reference		
NH Black	1.01	0.94–1.10	0.745
Hispanic	1.03	0.90–1.18	0.668
NH Other	0.87	0.78–0.97	0.015
T1 stage			
T1a	Reference		
T1b	9.12	8.30–10.02	< 0.001
T1c	22.25	20.28–24.43	< 0.001
Tumor grade			
Well differentiated	Reference		
Moderately differentiated	2.89	2.49–3.35	< 0.001
Poorly differentiated/undifferentiated	4.85	4.20–5.60	< 0.001
Histology			
Ductal	Reference		
Other histology	0.63	0.58–0.68	< 0.001
Surgery			
BCS	Reference		
Mastectomy	2.74	2.45–3.06	< 0.001
Radiation			
No	Reference		
Yes	3.87	3.49–4.28	< 0.001
Comorbidities			
CCI = 0	Reference		
CCI = 1	0.97	0.89–1.04	0.383
CCI = 2	0.70	0.60–0.81	< 0.001
CCI ≥ 3	0.60	0.48–0.74	< 0.001
Income			
< 46k	Reference		
46k–57k	1.01	0.92–1.13	0.741
58k–74k	1.11	1.004–1.24	0.041
> 74k	1.25	1.12–1.40	< 0.001
Unknown	1.49	0.81–2.83	0.209
Insurance			
Medicare/Medicaid	Reference		
Private	1.70	1.59–1.82	< 0.001
Uninsured	1.01	0.79–1.28	0.959
Other	1.34	1.10–1.65	0.004
No high‐school education			
> 15.3%	Reference		
9.1%–15.2%	1.002	0.91–1.10	0.962
5%–9%	0.99	0.90–1.10	0.973
< 5%	1.01	0.90–1.14	0.832
Unknown	0.78	0.41–1.42	0.424
Rurality			
Metro	Reference		
Urban–rural	1.14	1.05–1.24	0.003
Rurality unknown	1.14	0.94–1.40	0.183
Type of facility			
Community Ca. program	Reference		
Comprehensive Ca. center	0.97	0.87–1.08	0.587
Academic program	1.08	0.96–1.21	0.210
Integrated network CP	1.006	0.89–1.13	0.919
Year of diagnosis			
2010–2012	Reference		
2013–2015	1.20	1.12–1.29	< 0.001
2016–2019	1.28	1.20–1.37	< 0.001

Abbreviations: BCS, breast‐conserving surgery; Ca., cancer; CCI, Charlson‐Deyo Comorbidity Index; CI, confidence interval; CP, cancer program; k, thousand dollars; NA, not applicable; NH, non‐Hispanic; OR, odds ratio.

We also evaluated factors associated with chemotherapy use stratified by tumor size (Table S2). Among patients with T1c tumors, the odds of chemotherapy were lower for other histology (OR, 0.59; 95% CI, 0.53–0.66); however, such a difference was not seen in T1a tumors. Among patients with T1a tumors, comorbidities did not influence the odds of chemotherapy use; however, the odds of chemotherapy decreased in patients with comorbidities (OR, 0.46; 95% CI, 0.65–0.61) in patients with T1c tumors. Across all tumor sizes, older age adults had lower odds of chemotherapy, whereas private insurance increased the odds of chemotherapy use.

Further, we analyzed factors associated with chemotherapy use in different age groups (Table S3). In older age adults, individuals racialized as NH Black were associated with increased odds of chemotherapy use (OR, 1.26; 95% CI, 1.09–1.45), but this effect was not evidenced in young patients. Comorbidities did not influence the odds of chemotherapy use in young patients; however, the odds of chemotherapy decreased in older age patients with comorbidities (OR, 0.62; 95% CI, 0.45–0.86).

### 3.2 Benefit of Adjuvant Chemotherapy on Overall Survival in Patients With pT1N0M0 TNBC

**FIGURE 1:**
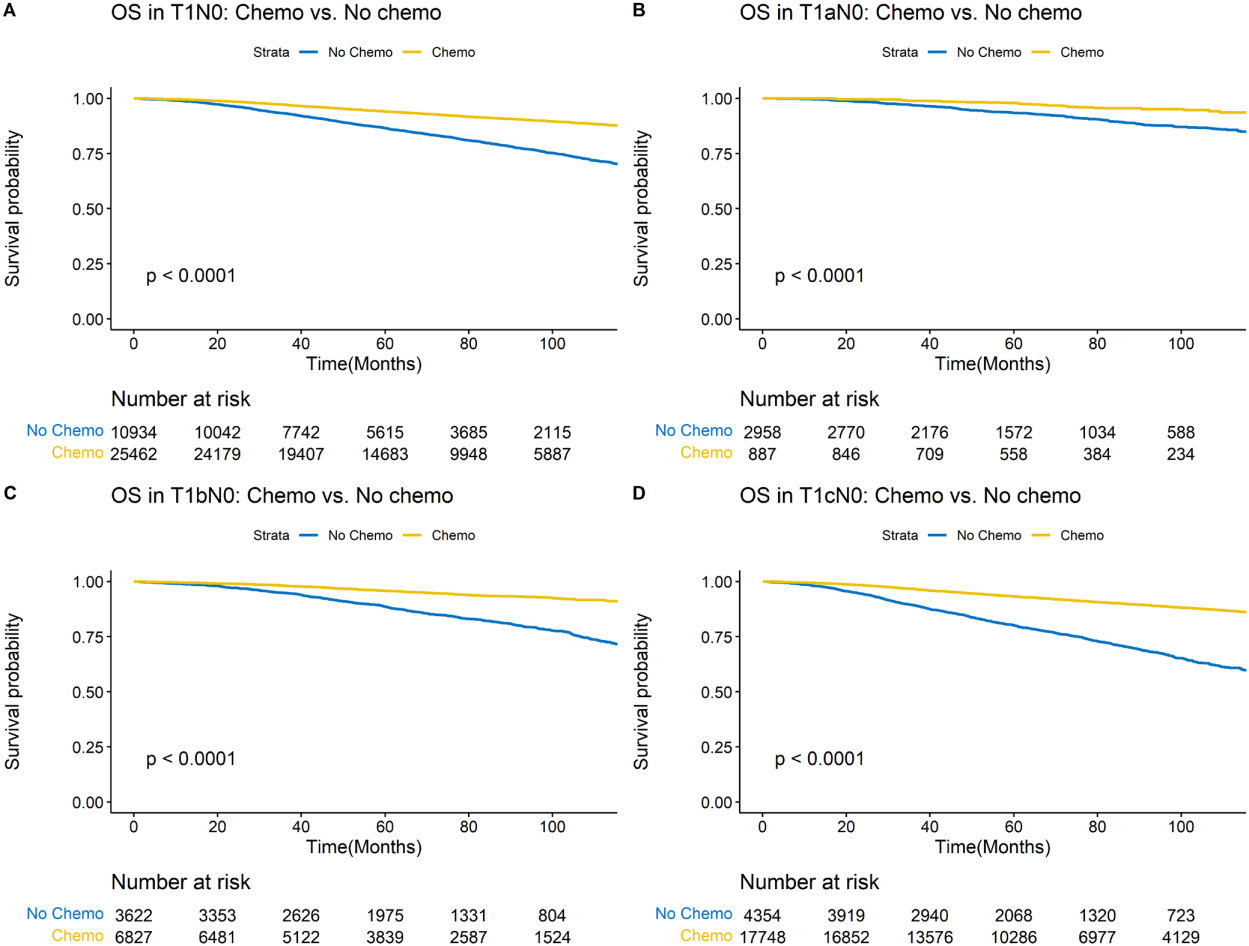
Overall survival in patients with pT1N0M0 triple‐negative breast cancer stratified by tumor size. (A) Overall survival in all patients; (B) Overall survival in patients with T1a tumors; (C) Overall survival in patients with T1b tumors; (D) Overall survival in patients with T1c tumors. TNBC, triple‐negative breast cancer.

After a median follow‐up of 66 months, 3766 (10%) patients with pT1N0M0 TNBC died. Kaplan–Meier curves revealed prolonged OS for the chemotherapy group as compared to the no chemotherapy group (*p* < 0.0001) (Figure 1). The 5‐year OS rate was 94% in patients treated with chemotherapy and 87% in patients who did not receive chemotherapy. Furthermore, the 5‐year OS rates for chemotherapy vs. no chemotherapy were 98% vs. 93%, 96% vs. 89%, and 93% vs. 80% for T1a, T1b, and T1c tumors, respectively. Additionally, adjuvant chemotherapy was associated with improved OS in patients who underwent mastectomy (5‐year OS rate, 94% vs. 84%; *p* < 0.001) and BCS (5‐year OS rate, 94% vs. 87%; *p* < 0.001) (Figure S4). In a multivariable model, chemotherapy was associated with improved OS (HR, 0.51; 95% CI, 0.47–0.55; *p* < 0.001). Other variables associated with improved OS included young age, Hispanic race, mastectomy, high household income, private insurance, and care facility other than community cancer program. Variables associated with worse OS included older age, NH Black, large tumor size, high tumor grade, and comorbidities (Table 3). The inverse propensity weighted analysis [29, 30, 31] revealed that chemotherapy was associated with improved OS at a similar magnitude (HR, 0.49; 95% CI, 0.44–0.54; *p* < 0.001) (Table S4), suggesting the robustness of the findings.

**TABLE 3:** Univariate and multivariable analysis of overall survival in patients with pT1N0M0 triple‐negative breast cancer.

Characteristics	Univariate			Multivariable		
	HR	95% CI	*p*	HR	95% CI	*p*
Chemotherapy						
No	Reference			Reference		
Yes	0.39	0.37–0.42	< 0.001	0.51	0.47–0.55	< 0.001
Age (years)						
50–70	Reference			Reference		
< 50	0.64	0.56–0.72	< 0.001	0.79	0.69–0.90	< 0.001
> 70	3.01	2.82–3.22	< 0.001	1.73	1.60–1.88	< 0.001
Race/ethnicity						
NH White	Reference			Reference		
NH Black	1.10	1.02–1.19	0.018	1.09	1.001–1.19	0.047
Hispanic	0.74	0.62–0.89	0.001	0.79	0.66–0.95	0.011
NH other	0.77	0.67–0.89	< 0.001	0.85	0.73–0.98	0.024
Radiation						
No	Reference			Reference		
Yes	0.68	0.64–0.73	< 0.001	0.61	0.55–0.68	< 0.001
Surgery type						
BCS	Reference			Reference		
Mastectomy	1.07	0.99–1.15	0.060	0.77	0.69–0.86	< 0.001
Rurality						
Metro	Reference			Reference		
Urban–rural	1.18	1.08–1.29	< 0.001	0.99	0.90–1.09	0.817
Unknown	0.84	0.66–1.07	0.150	0.91	0.71–1.16	0.440
T1 stage						
T1a	Reference			Reference		
T1b	1.21	1.06–1.39	0.004	1.45	1.26–1.66	< 0.001
T1c	1.58	1.40–1.79	< 0.001	2.16	1.90–2.46	< 0.001
Histology						
Ductal	Reference			Reference		
Other histology	0.99	0.90–1.10	0.910	0.91	0.82–1.007	0.067
Tumor grade						
Well differentiated	Reference			Reference		
Moderately differentiated	0.94	0.79–1.12	0.493	1.07	0.89–1.28	0.474
Poorly differentiated/undifferentiated	0.90	0.76–1.06	0.207	1.22	1.03–1.46	0.023
Comorbiditie**s**						
CCI < 2	Reference			Reference		
CCI ≥ 2	3.09	2.77–3.44	< 0.001	2.13	1.91–2.38	< 0.001
Income						
< 46k	Reference			Reference		
46 k–57 k	0.92	0.84–1.02	0.126	0.97	0.88–1.08	0.591
58 k–74 k	0.80	0.73–0.89	< 0.001	0.88	0.79–0.98	0.020
> 74k	0.59	0.54–0.65	< 0.001	0.73	0.66–0.81	< 0.001
Unknown	0.70	0.62–0.79	< 0.001	0.80	0.71–0.90	< 0.001
Insurance						
Medicaid/Medicare	Reference			Reference		
Private	0.35	0.33–0.37	< 0.001	0.61	0.57–0.67	< 0.001
Uninsured	0.43	0.31–0.60	< 0.001	0.69	0.50–0.95	0.022
Other	0.51	0.40–0.66	< 0.001	0.76	0.59–0.98	0.033
Facility						
Community Ca. program	Reference			Reference		
Comprehensive Ca. Center	0.82	0.73–0.92	0.001	0.88	0.78–0.99	0.038
Academic program	0.61	0.54–0.69	< 0.001	0.72	0.63–0.81	< 0.001
Integrated network CP	0.77	0.68–0.87	< 0.001	0.83	0.73–0.95	0.005
Unknown	0.29	0.22–0.39	< 0.001	0.62	0.46–0.83	0.001

Abbreviations: BCS, breast‐conserving surgery; Ca., cancer; CCI, Charlson‐Deyo Comorbidity Index; CP, cancer program; HR, hazard ratio; k, thousand dollars; NA, not applicable; NH, non‐Hispanic.

### 3.3 Subgroup Analysis

**FIGURE 2:**
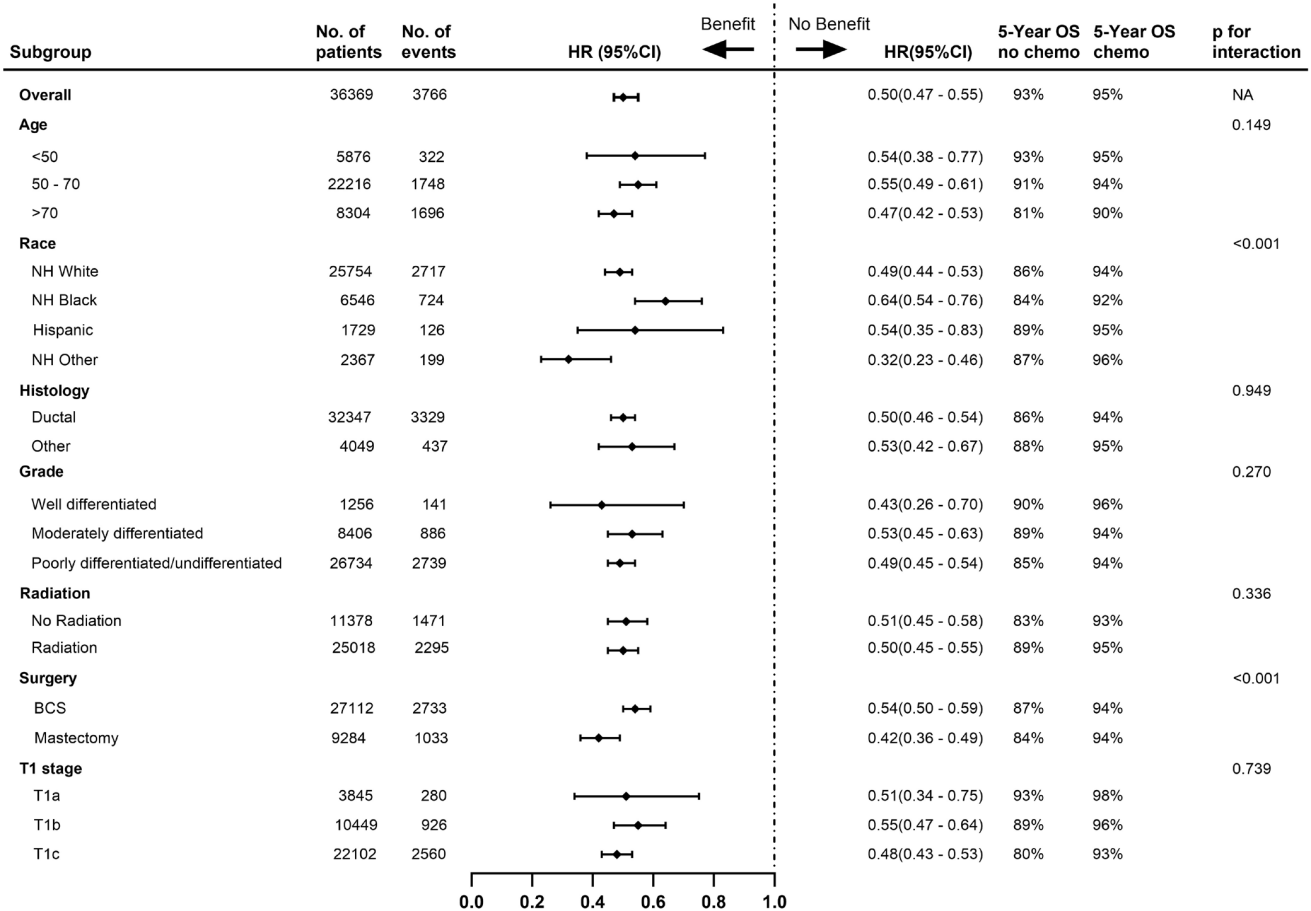
Benefit of chemotherapy in overall survival for pT1N0M0 triple‐negative breast cancer. Models were adjusted for race, age, comorbidities, tumor size, histology, tumor grade, radiation, type of surgery, household income, insurance, rurality, and type of treatment facility. BCS, breast‐conserving surgery; HR, hazard ratio; k, thousands; NH, non‐Hispanic; OS, overall survival.

We further analyzed the chemotherapy‐associated OS according to demographic, clinicopathological, and treatment factors (Figure 2). The association between chemotherapy and OS significantly varied by race and surgery type. The benefit of adjuvant chemotherapy was smaller in NH Black women (HR, 0.64; 95% CI, 0.54–0.76) as compared to NH White women (HR, 0.49; 95% CI, 0.44–0.53). Patients who underwent mastectomy (HR, 0.42; 95% CI, 0.36–0.49) had a larger chemotherapy benefit than those who underwent BCS (HR, 0.54; 95% CI, 0.50–0.59). Interestingly, the benefit of chemotherapy on OS was evidenced in all tumor size subgroups (HR for T1a, 0.51; HR for T1b, 0.55; HR for T1c, 0.48; P_interaction_ = 0.739). Additionally, the benefit of adjuvant chemotherapy in OS was similar in all age groups (P_interaction_ = 0.149), tumor histology (P_interaction_ = 0.949), tumor grade (P_interaction_ = 0.270), and radiotherapy (P_interaction_ = 0.336) subgroups.

### 3.4 Multiagent Chemotherapy in pT1N0M0 TNBC

We also evaluated the effect of single or multiagent chemotherapy in OS among patients with pT1N0M0 TNBC. In our study, 24,502 patients received multiagent chemotherapy and 960 received single‐agent chemotherapy (Tables S5 and S6). Compared to no chemotherapy, multiagent adjuvant chemotherapy (HR, 0.50; 95% CI, 0.46–0.54; *p* < 0.001) was associated with improved OS; whereas the administration of single‐agent chemotherapy (HR, 0.84; 95% CI, 0.63–1.12; *p* = 0.232) was not associated with OS (Table S7).

## 4 Discussion

To our knowledge, we report the largest study evaluating the benefit of adjuvant chemotherapy in pT1N0M0 TNBC. We showed that in patients with pT1N0M0 TNBC, adjuvant chemotherapy improved OS (HR, 0.51; 95% CI, 0.47–0.55; *p* < 0.001). The benefit of adjuvant chemotherapy in OS varied by race and surgery type; furthermore, this benefit was evidenced in T1a, T1b, and T1c tumors but with different absolute improvements in OS for each tumor size group.

A population study from the Netherlands included 1366 patients with T1N0M0 TNBC and reported that adjuvant chemotherapy was associated with improved OS (HR, 0.58; 95% CI, 0.46–0.73) and breast cancer‐specific survival (HR 0.65; 95% CI, 0.46–0.89), but the chemotherapy benefit was evidenced only in T1c tumors [32]. Of note, our study has a larger sample size and included patients from different ethnoracial backgrounds, which may explain some differences in the results. Additionally, they defined ER/PR as < 10% expression which may have led to the inclusion of some ER+ tumors which have better prognosis than TNBC tumors. Most studies evaluating the benefit of adjuvant chemotherapy in T1a/bN0 TNBC were small with few events [18, 20, 33, 34]. In a single institution study, An et al. reported the benefit of chemotherapy on relapse‐free survival in patients with T1N0M0 TNBC, but this benefit was evidenced only in T1c tumors [21]. Additionally, they conducted a meta‐analysis which revealed the benefit of adjuvant chemotherapy on the risk of disease recurrence in T1a/b tumors (Risk Ratio [RR], 0.60; 95% CI, 0.43–0.83); however, subgroup analysis revealed chemotherapy benefit in T1b tumors (RR, 0.62; 95% CI, 0.42–0.92) but not in T1a tumors (RR, 0.34; 95% CI, 0.31–1.33). It is noteworthy that T1a and T1b tumors had similar RR values, but the T1a group (*n* = 78, events = 8) had fewer events than the T1b (*n* = 406, events = 46) group, which could have affected the statistical analysis as suggested by the wide confidence intervals. Despite the similar level of relative benefit of adjuvant chemotherapy on OS that we found across all tumor sizes, the absolute benefit varies because the baseline OS rate differs. The improvement in 5‐year OS rate was larger in T1c tumors, with a 13% improvement (80% vs. 93%), whereas the benefit was 7% in T1b (89% vs. 96%) and 5% in T1a tumors (93% vs. 98%). Similar to other authors [35, 36, 37], our study also reports that patients with T1a tumors had excellent outcomes even without adjuvant chemotherapy. Unfortunately, we were unable to collect information about toxicities derived from chemotherapy use in our patients, especially for those with modest absolute benefit. The decision about adjuvant chemotherapy should be based on a comprehensive discussion about risks and benefits, but it should also involve patient comfort level with such benefits or risks since patients vary in the value they place on potential benefits of a toxic treatment such as chemotherapy [38, 39].

We found that compared to NH White women, NH Black women had the same odds of chemotherapy use but derived less benefit from chemotherapy. Prior gene expression analyses have demonstrated a higher prevalence of basal‐like phenotypes in NH Black women, which have a high mitotic index and marked nuclear pleomorphism, possibly accounting for differences in chemotherapy sensitivity and survival outcomes [40, 41]. Other factors that may contribute to these racial disparities include differences in tumor biology and tumor microenvironment, access to care, treatment delay, or other socio‐demographic factors [42, 43, 44]. Additionally, there might be differences in treatment side effects that result in racial differences in dose changes and treatment termination which may drive these racial disparities [45].

Age is a commonly used parameter in clinical practice to recommend adjuvant therapies. In our study, young patients had better OS, whereas older aged patients had worse OS. Despite the survival differences, old patients had lower odds of chemotherapy use. Our analysis was adjusted for Charlson‐Deyo comorbidity index, but there is literature suggesting that this score underestimates comorbidities [46]; therefore, unmeasured comorbidities may explain differences in chemotherapy use and OS in older patients. Moreover, comorbidities may increase the number of medication interactions with chemotherapy agents [47, 48] which could affect chemotherapy use and OS in older age patients. Additionally, it is possible that chemotherapy was not offered to older patients based on physician assumption for tolerance or that it was declined by patients [49]. It is important to highlight that our adjusted analysis revealed no difference in the benefit of adjuvant chemotherapy between old and young patients, supporting the rationale of using adjuvant chemotherapy in older patients without significant competing comorbidities.

Prior studies have demonstrated similar OS outcomes for mastectomy vs. BCS plus radiotherapy in patients with early breast cancer [50, 51]. In our study, after adjustment for radiation and other variables, patients who underwent mastectomy had better OS than those who underwent BCS. Patients who underwent mastectomy had higher odds of adjuvant chemotherapy and more benefit from chemotherapy. These results could be explained by unmeasured tumor risk factors, competing comorbidities, or confounding variables [52, 53]. Since mastectomy is a more aggressive surgery, there is a possibility of selection bias in which patients who underwent mastectomy had fewer unmeasured competing comorbidities than patients who underwent BCS, and the better OS found in patients with mastectomy is a reflection of better non‐breast cancer survival.

Our study has certain limitations. First, despite its large size, this is a retrospective study subjected to the biases of such studies. Second, the NCDB database does not provide information about the chemotherapy regimen, duration of chemotherapy, or dose reduction, which may affect chemotherapy efficacy. Third, we were unable to evaluate the use of immune checkpoint inhibitors or poly‐adenosine diphosphate ribose polymerase (PARP) inhibitors which have demonstrated improving survival outcomes in TNBC. Fourth, TNBC is a heterogeneous disease, and molecular profiling can distinguish different TNBC molecular subtypes which may have differences in chemotherapy benefit; however, this information was not available in the NCDB database. Fifth, studies have reported that patients with high levels of tumor‐infiltrating lymphocytes (TILs) have better survival following adjuvant chemotherapy [54]; however, information about TILs was not available in our database. Sixth, we were unable to assess the benefit of adjuvant chemotherapy on cancer‐specific survival since this information is not available in the NCDB database. Seventh, some subgroup analyses included few survival events. Lastly, we were unable to include individual socioeconomic variables in our analyses.

In conclusion, our study supports the value of adjuvant chemotherapy on OS for patients with pT1N0M0 TNBC, with the greatest magnitude of benefit observed in T1c tumors versus T1b and T1a tumors. Patient comfort level about prognosis and chemotherapy benefit is critical when deciding chemotherapy use for these patients. Future studies need to evaluate molecular biomarkers, tumor microenvironment, immune signaling pathways, and patient comorbidities and socioeconomic factors to identify subgroups that would derive the most chemotherapy benefit.

## Author Contributions

**Jesus D. Anampa:** conceptualization (equal), data curation (equal), formal analysis (equal), investigation (equal), methodology (equal), project administration (equal), resources (equal), software (equal), supervision (equal), validation (equal), visualization (equal), writing – original draft (equal), writing – review and editing (equal). **Alvaro Alvarez Soto:** data curation (equal), validation (equal), visualization (equal), writing – original draft (equal). **Rachel B. Jimenez:** conceptualization (equal), investigation (equal), visualization (equal), writing – original draft (equal), writing – review and editing (equal). **Samilia Obeng‐Gyasi:** methodology (equal), supervision (equal), visualization (equal), writing – original draft (equal), writing – review and editing (equal). **Xiaonan Xue:** data curation (equal), formal analysis (equal), methodology (equal), software (equal), supervision (equal), validation (equal), visualization (equal), writing – original draft (equal).

## Disclosure

Permission to reproduce material from other sources: The authors have created all materials in this manuscript (tables and figures).

## Ethics Statement

This study was approved by the Institutional Review Board (IRB) of the Albert Einstein College of Medicine. Due to the retrospective nature of this study, no informed consent was required.

## Conflicts of Interest

The authors declare no conflicts of interest.

## Supporting information


**Table S1:** Trends in chemotherapy use over time in patients with pT1N0M0 triple‐negative breast cancer. Chemo, chemotherapy; no chemo, no chemotherapy; n, number.
**Table S2:** Factors associated with the use of chemotherapy in patients with pT1N0M0 triple‐negative breast cancer stratified by tumor size. BCS, breast‐conserving surgery; Ca., cancer; CP, cancer program; CCI, Charlson‐Deyo Comorbidity Index; CI, confidence interval; diff., differentiated; k, thousand dollars; NA, not applicable; NH, non‐Hispanic; OR, odds ratio.
**Table S3:** Factors associated with the use of chemotherapy in patients with pT1N0M0 triple‐negative breast cancer stratified by age. BCS, breast‐conserving surgery; Ca., cancer; CP, cancer program; CCI, Charlson‐Deyo Comorbidity Index; CI, confidence interval; diff., differentiated; k, thousand dollars; NA, not applicable; NH, non‐Hispanic; OR, odds ratio.
**Table S4:** Multivariable analysis of overall survival after inverse propensity weighted analysis based on propensity score in patients with pT1N0M0 triple‐negative breast cancer. BCS, breast‐conserving surgery; Ca., cancer; CP, cancer program; CCI, Charlson‐Deyo Comorbidity Index; HR, hazard ratio; k, thousand dollars; NH, non‐Hispanic.
**Table S5:** Baseline characteristics of patients with pT1N0M0 tripe‐negative breast cancer comparing multiagent chemotherapy vs. no chemotherapy. BCS, breast‐conserving surgery; CCI, Charlson‐Deyo Comorbidity Index; IQR, interquartile range; k, thousand dollars; NH, non‐Hispanic; No., Number; TNBC, triple‐negative breast cancer; vs., versus; y, years.
**Table S6:** Factors associated with the use of multiagent chemotherapy in patients with pT1N0M0 triple‐negative breast cancer. BCS, breast‐conserving surgery; Ca., cancer; CP, cancer program; CCI, Charlson‐Deyo Comorbidity Index; CI, confidence interval; diff., differentiated; k, thousand dollars; NA, not applicable; NH, non‐Hispanic; OR, odds ratio.
**Table S7:** Univariate and multivariable analysis of overall survival with single or multi‐agent chemotherapy in patients with pT1N0M0 triple‐negative breast cancer. BCS, breast‐conserving surgery; Ca., cancer; CP, cancer program; CCI, Charlson‐Deyo Comorbidity Index; HR, hazard ratio; k, thousand dollars; NH, non‐Hispanic.
**Figure S1:** Strobe diagram of patients included for final analysis. HER2, human epidermal growth factor receptor 2; n, number; NCBD, National cancer database; TNBC, triple‐negative breast cancer.
**Figure S2:** Chemotherapy use by diagnosis year and age. (A) Chemotherapy use by diagnosis year; x axis represents year of diagnosis and y axis represents percentage of patients receiving chemotherapy. (B) Chemotherapy use by age; x axis represents age in years, y axis represents number of patients.
**Figure S3:** Chemotherapy use over time stratified by tumor size. X axis represents year of diagnosis, y axis represents percentage of patients.
**Figure S4:** Overall survival in patients with pT1N0M0 triple‐negative breast cancer stratified by surgery type. (A) Overall survival in patients with mastectomy; (B) Overall survival in patients with BCS. BCS, breast‐conserving surgery; TNBC, triple‐negative breast cancer.

## Data Availability

This study was conducted using data from the National Cancer Database (NCDB). A request was approved to use the participant user file data 2020; the download was completed on Mar 10, 2023. The spreadsheet created for the data analysis is available for the public after a request to the NCDB. The selection criteria necessary to obtain the matrix we used for this study are specified in the methods section of the manuscript.
