# Supplementary material for: Adjuvant Chemotherapy in Lymph Node‐Negative, T1 Triple‐Negative Breast Cancer
Source: Cancer Med. 2025 Nov 4;14(21):e71347. doi: 10.1002/cam4.71347 (PMC12585342; doi:10.1002/cam4.71347)
Supplement: Supplementary file 1 — Table S1: Trends in chemotherapy use over time in patients with pT1N0M0 triple‐negative breast cancer. Chemo, chemotherapy; no chemo, no chemotherapy; n, number. Table S2: Factors associated with the use of chemotherapy in patients with pT1N0M0 triple‐negative breast cancer stratified by tumor size. BCS, breast‐conserving surgery; Ca., cancer; CP, cancer program; CCI, Charlson‐Deyo Comorbidity Index; CI, confidence interval; diff., differentiated; k, thousand dollars; NA, not applicable; NH, non‐Hispanic; OR, odds ratio. Table S3: Factors associated with the use of chemotherapy in patients with pT1N0M0 triple‐negative breast cancer stratified by age. BCS, breast‐conserving surgery; Ca., cancer; CP, cancer program; CCI, Charlson‐Deyo Comorbidity Index; CI, confidence interval; diff., differentiated; k, thousand dollars; NA, not applicable; NH, non‐Hispanic; OR, odds ratio. Table S4: Multivariable analysis of overall survival after inverse propensity weighted analysis based on propensity score in patients with pT1N0M0 triple‐negative breast cancer. BCS, breast‐conserving surgery; Ca., cancer; CP, cancer program; CCI, Charlson‐Deyo Comorbidity Index; HR, hazard ratio; k, thousand dollars; NH, non‐Hispanic. Table S5: Baseline characteristics of patients with pT1N0M0 tripe‐negative breast cancer comparing multiagent chemotherapy vs. no chemotherapy. BCS, breast‐conserving surgery; CCI, Charlson‐Deyo Comorbidity Index; IQR, interquartile range; k, thousand dollars; NH, non‐Hispanic; No., Number; TNBC, triple‐negative breast cancer; vs., versus; y, years. Table S6: Factors associated with the use of multiagent chemotherapy in patients with pT1N0M0 triple‐negative breast cancer. BCS, breast‐conserving surgery; Ca., cancer; CP, cancer program; CCI, Charlson‐Deyo Comorbidity Index; CI, confidence interval; diff., differentiated; k, thousand dollars; NA, not applicable; NH, non‐Hispanic; OR, odds ratio. Table S7: Univariate and multivariable analysis of overall survival with s [file CAM4-14-e71347-s001.docx]

**Supplementary materials**

**Supplementary Table S1.** Trends in chemotherapy use over time in patients with pT1N0M0 triple-negative breast cancer

|  | **T1** | | **T1a** | | **T1b** | | **T1c** | |
| --- | --- | --- | --- | --- | --- | --- | --- | --- |
|  | **Chemo** | **No chemo** | **Chemo** | **No chemo** | **Chemo** | **No chemo** | **Chemo** | **No Chemo** |
| **Year** | **(n=25462)** | **(n=10934)** | **(n=887)** | **(n=2958)** | **(n=6827)** | **(n=3622)** | **(n=17748)** | **(n=4354)** |
| 2010 | 2462 (69%) | 1107 (31%) | 91 (25%) | 266 (75%) | 552 (59%) | 391 (41%) | 1819 (80%) | 450 (20%) |
| 2011 | 2691 (70%) | 1178 (30%) | 94 (25%) | 288 (75%) | 680 (60%) | 453 (40%) | 1917 (81%) | 437 (19%) |
| 2012 | 2544 (70%) | 1094 (30%) | 94 (27%) | 253 (73%) | 634 (64%) | 362 (36%) | 1816 (79%) | 479 (21%) |
| 2013 | 2610 (71%) | 1071 (29%) | 102 (26%) | 289 (74%) | 700 (67%) | 343 (33%) | 1808 (80%) | 439 (20%) |
| 2014 | 2616 (71%) | 1053 (29%) | 102 (27%) | 275 (73%) | 689 (66%) | 358 (34%) | 1825 (81%) | 420 (19%) |
| 2015 | 2643 (70%) | 1113 (30%) | 83 (21%) | 319 (79%) | 712 (67%) | 343 (33%) | 1848 (80%) | 451 (20%) |
| 2016 | 2501 (69%) | 1099 (31%) | 86 (20%) | 339 (80%) | 677 (66%) | 356 (34%) | 1738 (81%) | 404 (19%) |
| 2017 | 2546 (70%) | 1086 (30%) | 75 (19%) | 320 (81%) | 708 (69%) | 321 (31%) | 1763 (80%) | 445 (20%) |
| 2018 | 2487 (69%) | 1125 (31%) | 87 (22%) | 312 (78%) | 741 (67%) | 357 (33%) | 1659 (78%) | 456 (22%) |
| 2019 | 2362 (70%) | 1008 (30%) | 73 (20%) | 297 (80%) | 734 (68%) | 338 (32%) | 1555 (81%) | 373 (19%) |

**Supplementary Table S1.** Trends in chemotherapy use over time in patients with pT1N0M0 triple-negative breast cancer. Abbreviations: chemo, chemotherapy; no chemo, no chemotherapy; n, number.

**Supplementary Table S2.** Factors associated with the use of chemotherapy in patients with pT1N0M0 triple-negative breast cancer stratified by tumor size.

|  | **T1a** | | | **T1b** | | | **T1c** | | |
| --- | --- | --- | --- | --- | --- | --- | --- | --- | --- |
| **Characteristics** | **OR** | **95% CI** | **p-value** | **OR** | **95% CI** | **p-value** | **OR** | **95% CI** | **p-value** |
| **Age (years)** |  |  |  |  |  |  |  |  |  |
| 50-70 | Reference |  |  | Reference |  |  | Reference |  |  |
| Age < 50 | 1.24 | 0.97 – 1.58 | 0.073 | 2.01 | 1.66 – 2.45 | <0.001 | 1.69 | 1.45 – 1.99 | <0.001 |
| Age > 70 | 0.37 | 0.28 – 0.48 | <0.001 | 0.21 | 0.19 – 0.23 | <0.001 | 0.18 | 0.16 –0.19 | <0.001 |
| **Race/ethnicity** |  |  |  |  |  |  |  |  |  |
| NH White | Reference |  |  | Reference |  |  | Reference |  |  |
| NH Black | 0.99 | 0.79 – 1.26 | 0.980 | 1.06 | 0.93 – 1.21 | 0.393 | 0.98 | 0.88 – 1.10 | 0.760 |
| Hispanic | 1.12 | 0.76 – 1.63 | 0.559 | 1.08 | 0.84 – 1.38 | 0.557 | 0.99 | 0.83 – 1.20 | 0.969 |
| NH Other | 0.78 | 0.55 – 1.07 | 0..132 | 0.88 | 0.72 – 1.07 | 0.193 | 0.89 | 0.76 – 1.04 | 0.138 |
| **Tumor Grade** |  |  |  |  |  |  |  |  |  |
| Well diff. | Reference |  |  | Reference |  |  | Reference |  |  |
| Moderately diff. | 1.88 | 1.20 – 3.06 | 0.007 | 3.04 | 2.40 –3.86 | <0.001 | 3.07 | 2.50 – 3.77 | <0.001 |
| Poorly diff. | 3.49 | 2.26 – 5.63 | <0.001 | 4.85 | 3.86 – 6.12 | <0.001 | 5.20 | 4.26 – 6.34 | <0.001 |
| **Histology** |  |  |  |  |  |  |  |  |  |
| Ductal | Reference |  |  | Reference |  |  | Reference |  |  |
| Other histology | 0.84 | 0.63 – 1.12 | 0.253 | 0.64 | 0.55 – 0.74 | <0.001 | 0.59 | 0.53 – 0.66 | <0.001 |
| **Surgery** |  |  |  |  |  |  |  |  |  |
| BCS | Reference |  |  | Reference |  |  | Reference |  |  |
| Mastectomy | 1.78 | 1.17 – 2.77 | 0.008 | 2.45 | 1.99 – 3.00 | <0.001 | 2.81 | 2.45 – 3.22 | <0.001 |
| **Radiation** |  |  |  |  |  |  |  |  |  |
| No | Reference |  |  | Reference |  |  | Reference |  |  |
| Yes | 1.58 | 1.05 – 2.44 | 0.032 | 3.24 | 2.69 – 3.93 | <0.001 | 4.62 | 4.08 – 5.24 | <0.001 |
| **Comorbidities** |  |  |  |  |  |  |  |  |  |
| CCI = 0 | Reference |  |  | Reference |  |  | Reference |  |  |
| CCI = 1 | 0.89 | 0.69 – 1.12 | 0.325 | 0.95 | 0.84 – 1.09 | 0.489 | 0.99 | 0.89 – 1.10 | 0.824 |
| CCI = 2 | 0.55 | 0.28 – 0.99 | 0.063 | 0.73 | 0.56 – 0.96 | 0.021 | 0.69 | 0.57 – 0.84 | <0.001 |
| CCI ≥ 3 | 0.79 | 0.31 – 1.74 | 0.587 | 0.93 | 0.63 – 1.38 | 0.703 | 0.46 | 0.35 – 0.61 | <0.001 |
| **Income** |  |  |  |  |  |  |  |  |  |
| <46k | Reference |  |  | Reference |  |  | Reference |  |  |
| 46k – 57k | 0.96 | 0.70 – 1.31 | 0.773 | 0.95 | 0.80 – 1.13 | 0.544 | 1.06 | 0.92 – 1.21 | 0.419 |
| 58k – 74k | 0.99 | 0.72 – 1.37 | 0.962 | 1.05 | 0.88 – 1.26 | 0.598 | 1.17 | 1.02 – 1.36 | 0.030 |
| > 74k | 1.34 | 0.97 – 1.87 | 0.079 | 1.25 | 1.03 – 1.51 | 0.023 | 1.20 | 1.03 – 1.40 | 0.022 |
| Unknown | 2.24 | 0.31 – 11.18 | 0.354 | 3.46 | 1.18 – 12.24 | 0.034 | 0.82 | 0.39 – 1.84 | 0.618 |
| **Insurance** |  |  |  |  |  |  |  |  |  |
| Medicare/Medicaid | Reference |  |  | Reference |  |  | Reference |  |  |
| Private | 1.52 | 1.26 – 1.84 | <0.001 | 1.73 | 1.55 – 1.92 | <0.001 | 1.77 | 1.61 – 1.95 | <0.001 |
| Uninsured | 1.16 | 0.52 – 2.37 | 0.697 | 0.97 | 0.64 – 1.49 | 0.877 | 0.99 | 0.73 – 1.39 | 0.992 |
| Other | 1.57 | 0.92 – 2.62 | 0.088 | 1.06 | 0.75 – 1.50 | 0.755 | 1.50 | 1.13 – 2.01 | 0.005 |
| **No high-school ed.** |  |  |  |  |  |  |  |  |  |
| >15.3% | Reference |  |  | Reference |  |  | Reference |  |  |
| 9.1% - 15.2% | 0.81 | 0.61 – 1.07 | 0.137 | 0.99 | 0.85 – 1.16 | 0.907 | 1.06 | 0.93 – 1.20 | 0.363 |
| 5% - 9% | 0.86 | 0.64 – 1.16 | 0.334 | 0.99 | 0.84 – 1.18 | 0.935 | 1.04 | 0.91 – 1.20 | 0.570 |
| <5% | 0.77 | 0.55 – 1.07 | 0.120 | 1.01 | 0.83 – 1.23 | 0.883 | 1.09 | 0.93 – 1.28 | 0.295 |
| Unknown | 0.39 | 0.08 – 2.86 | 0.279 | 0.31 | 0.08 – 0.92 | 0.049 | 1.55 | 0.69 – 3.28 | 0.270 |
| **Rurality** |  |  |  |  |  |  |  |  |  |
| Metro | Reference |  |  | Reference |  |  | Reference |  |  |
| Urban-rural | 1.24 | 0.96 – 1.60 | 0.101 | 1.14 | 0.98 – 1.33 | 0.072 | 1.10 | 0.98 – 1.24 | 0.102 |
| Unknown | 1.81 | 1.11 – 2.90 | 0.015 | 1.15 | 0.83 – 1.60 | 0.401 | 0.96 | 0.73 – 1.27 | 0.762 |
| **Type of facility** |  |  |  |  |  |  |  |  |  |
| Community Ca. program | Reference |  |  | Reference |  |  | Reference |  |  |
| Comprehensive Ca. Center | 0.99 | 0.69 – 1.43 | 0.940 | 0.93 | 0.77 – 1.12 | 0.438 | 0.99 | 0.86 – 1.16 | 0.980 |
| Academic program | 0.98 | 0.68 – 1.42 | 0.902 | 1.17 | 0.96 – 1.42 | 0.109 | 1.03 | 0.88 – 1.21 | 0.686 |
| Integrated Network CP | 1.01 | 0.69 – 1.49 | 0.964 | 1.01 | 0.83 – 1.23 | 0.945 | 1.01 | 0.86 – 1.18 | 0.926 |
| Unknown | 1.39 | 0.77 – 2.49 | 0.273 | 0.81 | 0.53 – 1.25 | 0.330 | 1.47 | 1.04 – 2.10 | 0.032 |
| **Year of diagnosis** |  |  |  |  |  |  |  |  |  |
| 2010-2012 | Reference |  |  | Reference |  |  | Reference |  |  |
| 2013-2015 | 0.97 | 0.80 – 1.19 | 0.779 | 1.41 | 1.25 – 1.59 | <0.001 | 1.14 | 1.03 – 1.26 | 0.008 |
| 2016-2019 | 0.77 | 0.63 – 0.93 | 0.007 | 1.58 | 1.41 – 1.77 | <0.001 | 1.26 | 1.14 – 1.38 | <0.001 |

**Supplementary Table S2.** Factors associated with the use of chemotherapy in patients with pT1N0M0 triple-negative breast cancer stratified by tumor size. Abbreviations: BCS, breast-conserving surgery; Ca., cancer; CP, cancer program; CCI, Charlson-Deyo Comorbidity Index; CI, confidence interval; diff., differentiated; k, thousand dollars; NA, not applicable; NH, non-Hispanic; OR, odds ratio.

**Supplementary Table S3.** Factors associated with the use of chemotherapy in patients with pT1N0M0 triple-negative breast cancer stratified by age.

|  | **< 50 years old** | | | 1. **– 70 years old** | | | **> 70 years old** | | |
| --- | --- | --- | --- | --- | --- | --- | --- | --- | --- |
| **Characteristics** | **OR** | **95% CI** | **p-value** | **OR** | **95% CI** | **p-value** | **OR** | **95% CI** | **p-value** |
| **Race/ethnicity** |  |  |  |  |  |  |  |  |  |
| NH White | Reference |  |  | Reference |  |  | Reference |  |  |
| NH Black | 0.91 | 0.71 – 1.15 | 0.410 | 0.93 | 0.84 – 1.03 | 0.174 | 1.26 | 1.09 – 1.45 | 0.001 |
| Hispanic | 1.08 | 0.77 – 1.53 | 0.657 | 0.99 | 0.82 – 1.19 | 0.902 | 1.10 | 0.84 – 1.44 | 0.468 |
| NH Other | 0.93 | 0.67 – 1.29 | 0.636 | 0.85 | 0.73 – 0.98 | 0.026 | 0.93 | 0.75 – 1.14 | 0.487 |
| **Tumor Grade** |  |  |  |  |  |  |  |  |  |
| Well diff. | Reference |  |  | Reference |  |  | Reference |  |  |
| Moderately diff. | 5.09 | 3.27 – 7.92 | <0.001 | 3.44 | 2.87 – 4.13 | <0.001 | 1.48 | 1.14 – 1.94 | 0.003 |
| Poorly diff. | 8.59 | 5.68 – 12.98 | <0.001 | 6.07 | 5.09 – 7.25 | <0.001 | 2.32 | 1.80 – 3.02 | <0.001 |
| **T1 stage** |  |  |  |  |  |  |  |  |  |
| T1a | Reference |  |  |  |  |  |  |  |  |
| T1b | 12.30 | 9.69 – 15.67 | <0.001 | 9.49 | 8.50 –10.62 | <0.001 | 4.75 | 3.71 – 6.17 | <0.001 |
| T1c | 31.71 | 25.18 – 40.09 | <0.001 | 25.45 | 22.77 – 28.48 | <0.001 | 10.26 | 8.08 – 13.21 | <0.001 |
| **Histology** |  |  |  |  |  |  |  |  |  |
| Ductal | Reference |  |  | Reference |  |  | Reference |  |  |
| Other histology | 0.44 | 0.34 – 0.56 | <0.001 | 0.59 | 0.53 – 0.66 | <0.001 | 0.81 | 0.70 – 0.94 | 0.005 |
| **Surgery** |  |  |  |  |  |  |  |  |  |
| BCS | Reference |  |  | Reference |  |  | Reference |  |  |
| Mastectomy | 4.94 | 3.60 – 6.76 | <0.001 | 4.04 | 3.46 – 4.71 | <0.001 | 1.46 | 1.23 – 1.73 | <0.001 |
| **Radiation** |  |  |  |  |  |  |  |  |  |
| No | Reference |  |  | Reference |  |  | Reference |  |  |
| Yes | 7.41 | 5.41 – 10.10 | <0.001 | 5.47 | 4.75 – 6.30 | <0.001 | 2.20 | 1.90 – 2.55 | <0.001 |
| **Comorbidities** |  |  |  |  |  |  |  |  |  |
| CCI = 0 | Reference |  |  | Reference |  |  | Reference |  |  |
| CCI = 1 | 1.10 | 0.81 – 1.52 | 0.551 | 0.96 | 0.86 – 1.06 | 0.407 | 0.97 | 0.86 – 1.10 | 0.665 |
| CCI = 2 | 0.75 | 0.36 – 1.69 | 0.463 | 0.67 | 0.55 – 0.82 | <0.001 | 0.74 | 0.58 – 0.94 | 0.012 |
| CCI ≥ 3 | 0.69 | 0.22 – 2.62 | 0.543 | 0.56 | 0.42 – 0.76 | <0.001 | 0.62 | 0.45 – 0.86 | 0.003 |
| **Income** |  |  |  |  |  |  |  |  |  |
| 46k – 57k | 1.16 | 0.83 – 1.62 | 0.378 | 1.01 | 0.88 – 1.15 | 0.918 | 1.01 | 0.84 – 1.20 | 0.952 |
| 58k – 74k | 1.15 | 0.82 – 1.61 | 0.415 | 1.06 | 0.93 – 1.23 | 0.378 | 1.22 | 1.01 – 1.46 | 0.036 |
| > 74k | 1.31 | 0.92 – 1.86 | 0.139 | 1.24 | 1.07 – 1.44 | 0.004 | 1.28 | 1.05 – 1.56 | 0.013 |
| Unknown | 1.22 | 0.22 – 12.92 | 0.842 | 1.89 | 0.84 – 4.72 | 0.146 | 1.48 | 0.51 – 4.28 | 0.462 |
| **Insurance** |  |  |  |  |  |  |  |  |  |
| Medicare/Medicaid | Reference |  |  | Reference |  |  | Reference |  |  |
| Private | 1.78 | 1.39 – 2.25 | <0.001 | 1.83 | 1.69 – 1.97 | <0.001 | 1.21 | 1.04 – 1.41 | 0.016 |
| Uninsured | 1.63 | 0.92 – 3.01 | 0.105 | 1.01 | 0.76 – 1.35 | 0.964 | 0.52 | 0.21 – 1.22 | 0.144 |
| Other | 1.83 | 1.03 – 3.39 | 0.046 | 1.22 | 0.95 – 1.57 | 0.119 | 1.69 | 1.10 – 2.59 | 0.015 |
| **No high-school ed.** |  |  |  |  |  |  |  |  |  |
| >15.3% | Reference |  |  | Reference |  |  | Reference |  |  |
| 9.1% - 15.2% | 0.79 | 0.59 – 1.05 | 0.110 | 0.99 | 0.87 – 1.11 | 0.813 | 1.11 | 0.95 – 1.31 | 0.201 |
| 5% - 9% | 0.80 | 0.58 – 1.10 | 0.170 | 0.91 | 0.79 – 1.04 | 0.164 | 1.25 | 1.05 – 1.49 | 0.013 |
| <5% | 0.96 | 0.66 – 1.38 | 0.817 | 0.95 | 0.81 – 1.11 | 0.533 | 1.15 | 0.94 – 1.40 | 0.173 |
| Unknown | 0.83 | 0.08 – 4.72 | 0.858 | 0.54 | 0.22 – 1.23 | 0.165 | 0.97 | 0.33 – 2.81 | 0.955 |
| **Rurality** |  |  |  |  |  |  |  |  |  |
| Metro | Reference |  |  | Reference |  |  | Reference |  |  |
| Urban-rural | 1.39 | 1.04 – 1.89 | 0.030 | 1.02 | 0.91 – 1.14 | 0.790 | 1.26 | 1.08 – 1.45 | 0.001 |
| Unknown | 1.06 | 0.63 -1.86 | 0.845 | 1.10 | 0.85 – 1.44 | 0.475 | 1.25 | 0.88 – 1.77 | 0.199 |
| **Type of facility** |  |  |  |  |  |  |  |  |  |
| Community Ca. program | Reference |  |  | Reference |  |  | Reference |  |  |
| Comprehensive Ca. Center | 1.08 | 0.71 – 1.59 | 0.772 | 1.02 | 0.88 – 1.18 | 0.775 | 0.87 | 0.73 –1.04 | 0.137 |
| Academic program | 1.01 | 0.66 – 1.50 | 0.966 | 1.13 | 0.97 – 1.32 | 0.103 | 0.99 | 0.82 – 1.20 | 0.929 |
| Integrated Network CP | 1.10 | 0.71 – 1.68 | 0.667 | 1.04 | 0.89 – 1.21 | 0.655 | 0.93 | 0.76 – 1.13 | 0.454 |
| **Year of diagnosis** |  |  |  |  |  |  |  |  |  |
| 2010-2012 | Reference |  |  | Reference |  |  | Reference |  |  |
| 2013-2015 | 0.99 | 0.80 – 1.23 | 0.926 | 1.18 | 1.07 – 1.29 | <0.001 | 1.39 | 1.22 – 1.58 | <0.001 |
| 2016-2019 | 0.79 | 0.64 – 0.97 | 0.023 | 1.17 | 1.07 – 1.28 | <0.001 | 1.77 | 1.57 – 1.99 | <0.001 |

**Supplementary Table S3.** Factors associated with the use of chemotherapy in patients with pT1N0M0 triple-negative breast cancer stratified by age. Abbreviations: BCS, breast-conserving surgery; Ca., cancer; CP, cancer program; CCI, Charlson-Deyo Comorbidity Index; CI, confidence interval; diff., differentiated; k, thousand dollars; NA, not applicable; NH, non-Hispanic; OR, odds ratio.

**Supplementary Table S4.** Multivariable analysis of overall survival after inverse propensity weighted analysis based on propensity score in patients with pT1N0M0 triple-negative breast cancer.

|  | **Multivariable** | | |
| --- | --- | --- | --- |
| **Characteristics** | **HR** | **95% CI** | **p-value** |
| **Chemotherapy** |  |  |  |
| No | Reference |  |  |
| Yes | 0.49 | 0.44 – 0.54 | <0.001 |
| **Age (years)** |  |  |  |
| 50 – 70 | Reference |  |  |
| < 50 | 0.84 | 0.72 – 0.98 | 0.026 |
| >70 | 1.67 | 1.52 – 1.83 | <0.001 |
| **Race/ethnicity** |  |  |  |
| NH White | Reference |  |  |
| NH Black | 1.10 | 0.98 – 1.24 | 0.119 |
| Hispanic | 0.78 | 0.61 – 0.99 | 0.049 |
| NH other | 0.75 | 0.62 – 0.92 | <0.001 |
| **Radiation** |  |  |  |
| Not given | Reference |  |  |
| Yes | 0.69 | 0.56 – 0.85 | <0.001 |
| **Surgery type** |  |  |  |
| BCS | Reference |  |  |
| Mastectomy | 0.78 | 0.63 – 0.98 | 0.033 |
| **Rurality** |  |  |  |
| Metro | Reference |  |  |
| Urban-rural | 1.03 | 0.90 – 1.18 | 0.659 |
| Unknown | 0.91 | 0.66 – 1.25 | 0.544 |
| **T1 stage** |  |  |  |
| T1a | Reference |  |  |
| T1b | 1.40 | 1.21 – 1.62 | <0.001 |
| T1c | 2.30 | 2.00 – 2.63 | <0.001 |
| **Histology** |  |  |  |
| Ductal | Reference |  |  |
| Others | 0.85 | 0.75 – 0.98 | 0.025 |
| **Tumor Grade** |  |  |  |
| Well differentiated | Reference |  |  |
| Moderately differentiated | 1.16 | 0.95 – 1.41 | 0.144 |
| Poorly differentiated/Undifferentiated | 1.26 | 1.05 – 1.53 | 0.015 |
| **Comorbidities** |  |  |  |
| CCI <2 | Reference |  |  |
| CCI ≥2 | 2.08 | 1.80 – 2.41 | <0.001 |
| **Income** |  |  |  |
| <46k | Reference |  |  |
| 46k – 57k | 0.91 | 0.79 – 1.05 | 0.181 |
| 58k – 74k | 0.81 | 0.79 – 1.05 | 0.197 |
| >74k | 0.73 | 0.63 – 0.84 | <0.001 |
| Unknown | 0.84 | 0.71 – 0.99 | 0.046 |
| **Insurance** |  |  |  |
| Medicaid/Medicare | Reference |  |  |
| Private | 0.58 | 0.52 – 0.65 | <0.001 |
| Uninsured | 0.71 | 0.47 – 1.08 | 0.106 |
| Other | 0.63 | 0.45 – 0.88 | 0.007 |
| **Facility** |  |  |  |
| Community Ca. program | Reference |  |  |
| Comprehensive Ca. Center | 0.93 | 0.79 – 1.10 | 0.407 |
| Academic program | 0.81 | 0.69 – 0.97 | 0.019 |
| Integrated Network CP | 0.92 | 0.77 – 1.10 | 0.359 |
| Unknown | 0.72 | 0.52 – 1.01 | 0.057 |

**Supplementary Table S4.** Multivariable analysis of overall survival after inverse propensity weighted analysis based on propensity score in patients with pT1N0M0 triple-negative breast cancer. Abbreviations: BCS, breast-conserving surgery; Ca., cancer; CP, cancer program; CCI, Charlson-Deyo Comorbidity Index; HR, hazard ratio; k, thousand dollars; NH, non-Hispanic.

**Supplementary Table S5.** Baseline characteristics of patients with pT1N0M0 tripe-negative breast cancer comparing multiagent chemotherapy vs. no chemotherapy.

|  | **Overall** | **Multiagent chemo** | **No Chemo** |  |
| --- | --- | --- | --- | --- |
| **Characteristics** | **(n=35436)** | **(n=24502)** | **(n=10934)** | **p-value** |
| **Age, y, median, IQR** | 62 (53 – 70) | 59 (51 – 66) | 69 (61 – 76) |  |
| **Age, No. (%)** |  |  |  | <0.001 |
| < 50 | 5718 (16%) | 4965 (20%) | 753 (7%) |  |
| 50-70 | 21649 (61%) | 16505 (67%) | 5144 (47%) |  |
| >70 | 8069 (23%) | 3032 (12%) | 5037 (46%) |  |
| **Year of Diagnosis** |  |  |  | 0.043 |
| 2010-2012 | 10760 (30%) | 7381 (30%) | 3379 (31%) |  |
| 2013-2015 | 10811 (31%) | 7574 (31%) | 3237 (30%) |  |
| 2016-2019 | 13865 (39%) | 9547 (39%) | 4318 (39%) |  |
| **Race/ethnicity** |  |  |  | <0.001 |
| NH White | 25102 (71%) | 17161 (70%) | 7941 (73%) |  |
| NH Black | 6384 (18%) | 4572 (19%) | 1812 (17%) |  |
| Hispanics | 1653 (5%) | 1199 (5%) | 454 (4%) |  |
| NH Other | 2297 (6%) | 1570 (6%) | 727 (7%) |  |
| **Histology** |  |  |  | <0.001 |
| Ductal | 31474 (89%) | 22184 (91%) | 9290 (85%) |  |
| Lobular | 274 (1%) | 129 (1%) | 145 (1%) |  |
| Ductolobular | 275 (1%) | 175 (1%) | 100 (1%) |  |
| Other | 3413 (10%) | 2014 (8%) | 1399 (13%) |  |
| **Tumor Grade** |  |  |  | <0.001 |
| Well differentiated | 1243 (4%) | 419 (2%) | 824 (8%) |  |
| Moderately differentiated | 8200 (23%) | 4521 (18%) | 3679 (34%) |  |
| Poorly differentiated/Undifferentiated | 25993 (73%) | 19562 (80%) | 6431 (59%) |  |
| **Type of facility** |  |  |  | <0.001 |
| Community cancer program | 2527 (7%) | 1688 (7%) | 839 (8%) |  |
| Comprehensive cancer program | 14106 (40%) | 9497 (39%) | 4609 (42%) |  |
| Academic program | 10259 (29%) | 7259 (30%) | 3000 (27%) |  |
| Integrated Network cancer program | 7246 (20%) | 4901 (20%) | 2345 (21%) |  |
| Unknown | 1298 (4%) | 1157 (5%) | 141 (1%) |  |
| **Geographic Location** |  |  |  | <0.001 |
| New England | 2168 (6%) | 1515 (6%) | 653 (6%) |  |
| Middle Atlantic | 5349 (15%) | 3855 (16%) | 1494 (14%) |  |
| South Atlantic | 8125 (23%) | 5512 (22%) | 2613 (24%) |  |
| East North Central | 6170 (17%) | 4265 (17%) | 1905 (17%) |  |
| East South Central | 2525 (7%) | 1726 (7%) | 799 (7%) |  |
| West North Central | 2529 (7%) | 1700 (7%) | 829 (8%) |  |
| West South Central | 2452 (7%) | 1593 (7%) | 859 (8%) |  |
| Mountain | 1428 (4%) | 964 (4%) | 464 (4%) |  |
| Pacific | 3392 (10%) | 2215 (9%) | 1177 (11%) |  |
| Unknown | 1298 (4%) | 1157 (5%) | 141 (1%) |  |
| **Rurality** |  |  |  | 0.082 |
| Metro | 29858 (84%) | 20585 (84%) | 9273 (85%) |  |
| Urban-rural | 4809 (13%) | 3363 (14%) | 1446 (13%) |  |
| Unknown | 769 (2%) | 554 (2%) | 215 (2%) |  |
| **Insurance** |  |  |  | <0.001 |
| Medicaid/Medicare | 15805 (45%) | 8854 (36%) | 6951 (64%) |  |
| Private | 18458 (52%) | 14788 (60%) | 3670 (34%) |  |
| Uninsured | 466 (1%) | 343 (1%) | 123 (1%) |  |
| Other | 707 (2%) | 517 (2%) | 190 (2%) |  |
| **Income** |  |  |  | <0.001 |
| <46k | 5021 (14%) | 3362 (14%) | 1659 (15%) |  |
| 46k – 57k | 6423 (18%) | 4363 (18%) | 2060 (19%) |  |
| 58k – 74k | 7285 (21%) | 5004 (20%) | 2281 (21%) |  |
| >74k | 11834 (33%) | 8378 (34%) | 3456 (32%) |  |
| Unknown | 4873 (14%) | 3395 (14%) | 1478 (14%) |  |
| **No high-school education** |  |  |  | 0.064 |
| >15.3% | 5910 (17%) | 4046 (17%) | 1864 (17%) |  |
| 9.1% – 15.2% | 8474 (24%) | 5812 (24%) | 2662 (24%) |  |
| 5% – 9% | 8989 (25%) | 6188 (25%) | 2801 (26%) |  |
| <5% | 7264 (20%) | 5114 (21%) | 2150 (20%) |  |
| Unknown | 4799 (14%) | 3342 (14%) | 1457 (13%) |  |
| **Comorbidities** |  |  |  | <0.001 |
| CCI = 0 | 28942 (82%) | 20365 (83%) | 8577 (78%) |  |
| CCI = 1 | 5004 (14%) | 3311 (14%) | 1693 (15%) |  |
| CCI = 2 | 1020 (3%) | 586 (2%) | 434 (4%) |  |
| CCI > 3 | 470 (1%) | 240 (1%) | 230 (2%) |  |
| **Radiation** |  |  |  | <0.001 |
| No | 11020 (31%) | 6956 (28%) | 4064 (37%) |  |
| Yes | 24416 (69%) | 17546 (72%) | 6870 (63%) |  |
| **Surgery type** |  |  |  | 0.097 |
| BCS | 26409 (75%) | 18197 (74%) | 8212 (75%) |  |
| Mastectomy | 9027 (25%) | 6305 (26%) | 2722 (25%) |  |

**Supplementary Table S5.** Baseline characteristics of patients with pT1N0M0 tripe-negative breast cancer comparing multiagent chemotherapy vs. no chemotherapy. Abbreviations: BCS, breast-conserving surgery; CCI, Charlson-Deyo Comorbidity Index; IQR, interquartile range; k, thousand dollars; NH, non-Hispanic; No., Number; TNBC, triple-negative breast cancer; vs, versus; y, years.

**Supplementary Table S6.** Factors associated with the use of multiagent chemotherapy in patients with pT1N0M0 triple-negative breast cancer.

|  | **All** | | | **Mastectomy** | | | **BCS** | | |
| --- | --- | --- | --- | --- | --- | --- | --- | --- | --- |
| **Characteristics** | **OR** | **95% CI** | **p-value** | **OR** | **95% CI** | **p-value** | **OR** | **95% CI** | **p-value** |
| **Age (years)** |  |  |  |  |  |  |  |  |  |
| 50-70 | Reference |  |  | Reference |  |  | Reference |  |  |
| Age < 50 | 1.71 | 1.53 – 1.90 | <0.001 | 1.58 | 1.34 – 1.88 | <0.001 | 1.70 | 1.48 – 1.96 | <0.001 |
| Age > 70 | 0.18 | 0.17 – 0.20 | <0.001 | 0.16 | 0.13 – 0.18 | <0.001 | 0.19 | 0.18 – 0.21 | <0.001 |
| **Race/ethnicity** |  |  |  |  |  |  |  |  |  |
| NH White | Reference |  |  | Reference |  |  | Reference |  |  |
| NH Black | 1.02 | 0.95 – 1.11 | 0.564 | 1.01 | 0.85 – 1.20 | 0.849 | 1.04 | 0.95 – 1.13 | 0.450 |
| Hispanics | 0.99 | 0.86 – 1.14 | 0.875 | 0.73 | 0.56 – 0.94 | 0.015 | 1.14 | 0.96 – 1.35 | 0.135 |
| NH Other | 0.86 | 0.77 – 0.97 | 0.010 | 0.74 | 0.60 – 0.91 | 0.004 | 0.93 | 0.81 – 1.06 | 0.270 |
| **T1 stage** |  |  |  |  |  |  |  |  |  |
| T1a | Reference |  |  | Reference |  |  | Reference |  |  |
| T1b | 9.57 | 8.70–10.55 | <0.001 | 7.82 | 6.53 – 9.40 | <0.001 | 10.51 | 9.37 – 11.79 | <0.001 |
| T1c | 23.90 | 21.73 – 26.31 | <0.001 | 18.03 | 15.15 – 21.52 | <0.001 | 27.19 | 24.27 – 30.51 | <0.001 |
| **Tumor Grade** |  |  |  |  |  |  |  |  |  |
| Well diff. | Reference |  |  | Reference |  |  | Reference |  |  |
| Moderately diff. | 2.86 | 2.46 – 3.32 | <0.001 | 2.53 | 1.85 – 3.48 | <0.001 | 2.95 | 2.49 – 3.51 | <0.001 |
| Poorly diff. | 4.83 | 4.18 – 5.59 | <0.001 | 3.94 | 2.91 – 5.35 | <0.001 | 5.16 | 4.38 – 6.10 | <0.001 |
| **Histology** |  |  |  |  |  |  |  |  |  |
| Ductal | Reference |  |  | Reference |  |  | Reference |  |  |
| Others | 0.63 | 0.58 – 0.69 | <0.001 | 0.65 | 0.55 – 0.78 | <0.001 | 0.62 | 0.56 – 0.69 | <0.001 |
| **Surgery** |  |  |  |  |  |  |  |  |  |
| BCS | Reference |  |  | NA |  |  | NA |  |  |
| Mastectomy | 2.99 | 2.67 – 3.35 | <0.001 | NA | NA | NA | NA | NA | NA |
| **Radiation** |  |  |  |  |  |  |  |  |  |
| Not given | Reference |  |  | Reference |  |  | Reference |  |  |
| Yes | 4.28 | 3.85 – 4.76 | <0.001 | 2.17 | 1.48 – 3.22 | <0.001 | 4.62 | 4.14 – 5.16 | <0.001 |
| **Comorbidities** |  |  |  |  |  |  |  |  |  |
| CCI = 0 | Reference |  |  | Reference |  |  | Reference |  |  |
| CCI = 1 | 0.96 | 0.89 – 1.04 | 0.368 | 0.86 | 0.73 – 1.003 | 0.055 | 1.001 | 0.91 – 1.10 | 0.978 |
| CCI = 2 | 0.68 | 0.58 – 0.79 | <0.001 | 0.60 | 0.44 – 0.83 | 0.002 | 0.69 | 0.58 – 0.83 | <0.001 |
| CCI ≥ 3 | 0.59 | 0.47 – 0.73 | <0.001 | 0.42 | 0.26 – 0.67 | <0.001 | 0.64 | 0.50 – 0.83 | <0.001 |
| **Income** |  |  |  |  |  |  |  |  |  |
| <46k | Reference |  |  | Reference |  |  | Reference |  |  |
| 46k – 57k | 1.02 | 0.92 – 1.13 | 0.679 | 0.99 | 0.81 – 1.22 | 0.986 | 1.02 | 0.90 – 1.15 | 0.757 |
| 58k – 74k | 1.13 | 1.01 – 1.26 | 0.027 | 1.16 | 0.93 – 1.44 | 0.168 | 1.11 | 0.98 – 1.26 | 0.098 |
| > 74k | 1.24 | 1.11 – 1.39 | <0.001 | 1.25 | 0.99 – 1.58 | 0.050 | 1.23 | 1.08 – 1.40 | 0.002 |
| Unknown | 1.53 | 0.83 – 2.93 | 0.186 | 2.19 | 0.70 – 7.73 | 0.194 | 1.30 | 0.63 – 2.84 | 0.495 |
| **Insurance** |  |  |  |  |  |  |  |  |  |
| Medicare/Medicaid | Reference |  |  | Reference |  |  | Reference |  |  |
| Private | 1.72 | 1.61 – 1.84 | <0.001 | 1.79 | 1.56 – 2.04 | <0.001 | 1.69 | 1.57 – 1.83 | <0.001 |
| Uninsured | 1.01 | 0.79 – 1.29 | 0.961 | 1.69 | 1.004 – 2.97 | 0.056 | 0.86 | 0.66 – 1.14 | 0.293 |
| Other | 1.31 | 1.07 – 1.62 | 0.009 | 1.29 | 0.87 – 1.95 | 0.201 | 1.31 | 1.03 – 1.67 | 0.028 |
| **No high-school ed.** |  |  |  |  |  |  |  |  |  |
| >15.3% | Reference |  |  | Reference |  |  | Reference |  |  |
| 9.1% - 15.2% | 1.001 | 0.91 – 1.10 | 0.987 | 1.19 | 0.99 – 1.44 | 0.058 | 0.94 | 0.85 – 1.05 | 0.302 |
| 5% - 9% | 1.002 | 0.90 – 1.11 | 0.957 | 1.03 | 0.84 – 1.27 | 0.711 | 0.99 | 0.88 – 1.11 | 0.853 |
| <5% | 1.04 | 0.92 – 1.16 | 0.565 | 1.07 | 0.85 – 1.36 | 0.527 | 1.02 | 0.89 – 1.17 | 0.805 |
| Unknown | 0.77 | 0.40 – 1.42 | 0.418 | 0.56 | 0.15 – 1.77 | 0.346 | 0.88 | 0.40 – 1.82 | 0.744 |
| **Rurality** |  |  |  |  |  |  |  |  |  |
| Metro | Reference |  |  | Reference |  |  | Reference |  |  |
| Urban-rural | 1.16 | 1.06 – 1.27 | <0.001 | 1.19 | 1.01 – 1.40 | 0.034 | 1.15 | 1.03 – 1.27 | 0.009 |
| Unknown | 1.14 | 0.93 – 1.39 | 0.204 | 1.34 | 0.89 – 2.07 | 0.162 | 1.08 | 0.86 – 1.36 | 0.520 |
| **Type of facility** |  |  |  |  |  |  |  |  |  |
| Community Ca. program | Reference |  |  | Reference |  |  | Reference |  |  |
| Comprehensive Ca. Center | 0.97 | 0.87 – 1.09 | 0.612 | 1.10 | 0.88 – 1.38 | 0.386 | 0.92 | 0.81 – 1.05 | 0.236 |
| Academic program | 1.08 | 0.97 – 1.22 | 0.171 | 1.48 | 1.17 – 1.88 | 0.001 | 0.96 | 0.84 – 1.10 | 0.605 |
| Integrated Network CP | 1.02 | 0.91 – 1.16 | 0.676 | 1.13 | 0.89 – 1.45 | 0.293 | 0.98 | 0.85 – 1.13 | 0.813 |
| Unknown | 1.25 | 0.98 – 1.60 | 0.070 | 1.43 | 1.01 – 2.03 | 0.039 | 1.22 | 0.83 – 1.81 | 0.320 |
| **Year of diagnosis** |  |  |  |  |  |  |  |  |  |
| 2010-2012 | Reference |  |  | Reference |  |  | Reference |  |  |
| 2013-2015 | 1.21 | 1.12 – 1.30 | <0.001 | 1.12 | 0.98 – 1.29 | 0.090 | 1.24 | 1.14 – 1.35 | <0.001 |
| 2016-2019 | 1.29 | 1.21 – 1.39 | <0.001 | 1.11 | 0.97 – 1.28 | 0.101 | 1.36 | 1.25 – 1.47 | <0.001 |

**Supplementary Table S6.** Factors associated with the use of multiagent chemotherapy in patients with pT1N0M0 triple-negative breast cancer. Abbreviations: BCS, breast-conserving surgery; Ca., cancer; CP, cancer program; CCI, Charlson-Deyo Comorbidity Index; CI, confidence interval; diff., differentiated; k, thousand dollars; NA, not applicable; NH, non-Hispanic; OR, odds ratio.

**Supplementary Table S7.** Univariate and multivariable analysis of overall survival with single or multiagent chemotherapy in patients with pT1N0M0 triple-negative breast cancer.

|  | **Univariate** | | | **Multivariable** | | |
| --- | --- | --- | --- | --- | --- | --- |
| **Characteristics** | **HR** | **95% CI** | **p-value** | **HR** | **95% CI** | **p-value** |
| **Chemotherapy** |  |  |  |  |  |  |
| No | Reference |  |  | Reference |  |  |
| Single agent | 0.83 | 0.62 – 1.10 | 0.184 | 0.84 | 0.63 – 1.12 | 0.232 |
| Multiagent | 0.38 | 0.36 – 0.41 | <0.001 | 0.50 | 0.46 – 0.54 | <0.001 |
| Unknown regimen | 0.39 | 0.29 – 0.53 | <0.001 | 0.45 | 0.34 – 0.61 | <0.001 |
| **Age (years)** |  |  |  |  |  |  |
| 50 – 70 | Reference |  |  | Reference |  |  |
| < 50 | 0.64 | 0.56 – 0.72 | <0.001 | 0.79 | 0.69 – 0.90 | <0.001 |
| >70 | 3.01 | 2.82 – 3.22 | <0.001 | 1.72 | 1.58 – 1.86 | <0.001 |
| **Race/ethnicity** |  |  |  |  |  |  |
| NH White | Reference |  |  | Reference |  |  |
| NH Black | 1.10 | 1.02 – 1.20 | 0.018 | 1.10 | 1.003 – 1.19 | 0.041 |
| Hispanic | 0.74 | 0.62 – 0.89 | 0.001 | 0.79 | 0.66 – 0.95 | 0.011 |
| NH other | 0.77 | 0.67 – 0.89 | <0.001 | 0.85 | 0.73 – 0.98 | 0.023 |
| **Radiation** |  |  |  |  |  |  |
| No | Reference |  |  | Reference |  |  |
| Yes | 0.68 | 0.64 – 0.72 | <0.001 | 0.61 | 0.55 – 0.68 | <0.001 |
| **Surgery type** |  |  |  |  |  |  |
| BCS | Reference |  |  | Reference |  |  |
| Mastectomy | 1.07 | 0.99 – 1.15 | 0.060 | 0.77 | 0.69 – 0.86 | <0.001 |
| **Rurality** |  |  |  |  |  |  |
| Metro | Reference |  |  | Reference |  |  |
| Urban-rural | 1.18 | 1.08 – 1.29 | <0.001 | 0.99 | 0.90 – 1.08 | 0.821 |
| Unknown | 0.84 | 0.66 – 1.07 | 0.150 | 0.90 | 0.72 – 1.15 | 0.440 |
| **T1 stage** |  |  |  |  |  |  |
| T1a | Reference |  |  | Reference |  |  |
| T1b | 1.21 | 1.06 – 1.39 | 0.004 | 1.44 | 1.26 – 1.65 | <0.001 |
| T1c | 1.58 | 1.40 – 1.79 | <0.001 | 2.17 | 1.90 – 2.46 | <0.001 |
| **Histology** |  |  |  |  |  |  |
| Ductal | Reference |  |  | Reference |  |  |
| Other histology | 0.99 | 0.90 – 1.10 | 0.910 | 0.91 | 0.82 – 1.01 | 0.068 |
| **Tumor Grade** |  |  |  |  |  |  |
| Well differentiated | Reference |  |  | Reference |  |  |
| Moderately differentiated | 0.94 | 0.79 – 1.12 | 0.493 | 1.07 | 0.89 – 1.28 | 0.478 |
| Poorly differentiated/Undifferentiated | 0.90 | 0.76 – 1.06 | 0.207 | 1.22 | 1.03 – 1.46 | 0.023 |
| **Comorbidities** |  |  |  |  |  |  |
| CCI <2 | Reference |  |  | Reference |  |  |
| CCI ≥2 | 3.09 | 2.77 – 3.44 | <0.001 | 2.12 | 1.90 – 2.37 | <0.001 |
| **Income** |  |  |  |  |  |  |
| <46k | Reference |  |  | Reference |  |  |
| 46k – 57k | 0.92 | 0.84 – 1.02 | 0.126 | 0.97 | 0.88 – 1.08 | 0.597 |
| 58k – 74k | 0.80 | 0.73 – 0.89 | <0.001 | 0.88 | 0.79 – 0.98 | 0.021 |
| >74k | 0.59 | 0.54 – 0.65 | <0.001 | 0.74 | 0.66 – 0.82 | <0.001 |
| Unknown | 0.70 | 0.62 – 0.79 | <0.001 | 0.80 | 0.71 – 0.91 | <0.001 |
| **Insurance** |  |  |  |  |  |  |
| Medicaid/Medicare | Reference |  |  | Reference |  |  |
| Private | 0.35 | 0.33 – 0.37 | <0.001 | 0.62 | 0.57 – 0.67 | <0.001 |
| Uninsured | 0.43 | 0.31 – 0.60 | <0.001 | 0.68 | 0.50 – 0.95 | 0.021 |
| Other | 0.51 | 0.40 – 0.66 | <0.001 | 0.76 | 0.59 – 0.98 | 0.033 |
| **Facility** |  |  |  |  |  |  |
| Community Ca. program | Reference |  |  | Reference |  |  |
| Comprehensive Ca. Center | 0.82 | 0.73 – 0.92 | 0.001 | 0.88 | 0.78 – 0.99 | 0.033 |
| Academic program | 0.61 | 0.54 – 0.69 | <0.001 | 0.71 | 0.63 – 0.81 | <0.001 |
| Integrated Network CP | 0.77 | 0.68 – 0.87 | <0.001 | 0.83 | 0.73 – 0.95 | 0.005 |
| Unknown | 0.29 | 0.22 – 0.39 | <0.001 | 0.61 | 0.45 – 0.83 | 0.001 |

**Supplementary Table S7.** Univariate and multivariable analysis of overall survival with single or multi-agent chemotherapy in patients with pT1N0M0 triple-negative breast cancer. Abbreviations: BCS, breast-conserving surgery; Ca., cancer; CP, cancer program; CCI, Charlson-Deyo Comorbidity Index; HR, hazard ratio; k, thousand dollars; NH, non-Hispanic.

**Supplementary Figure S1.** Strobe diagram


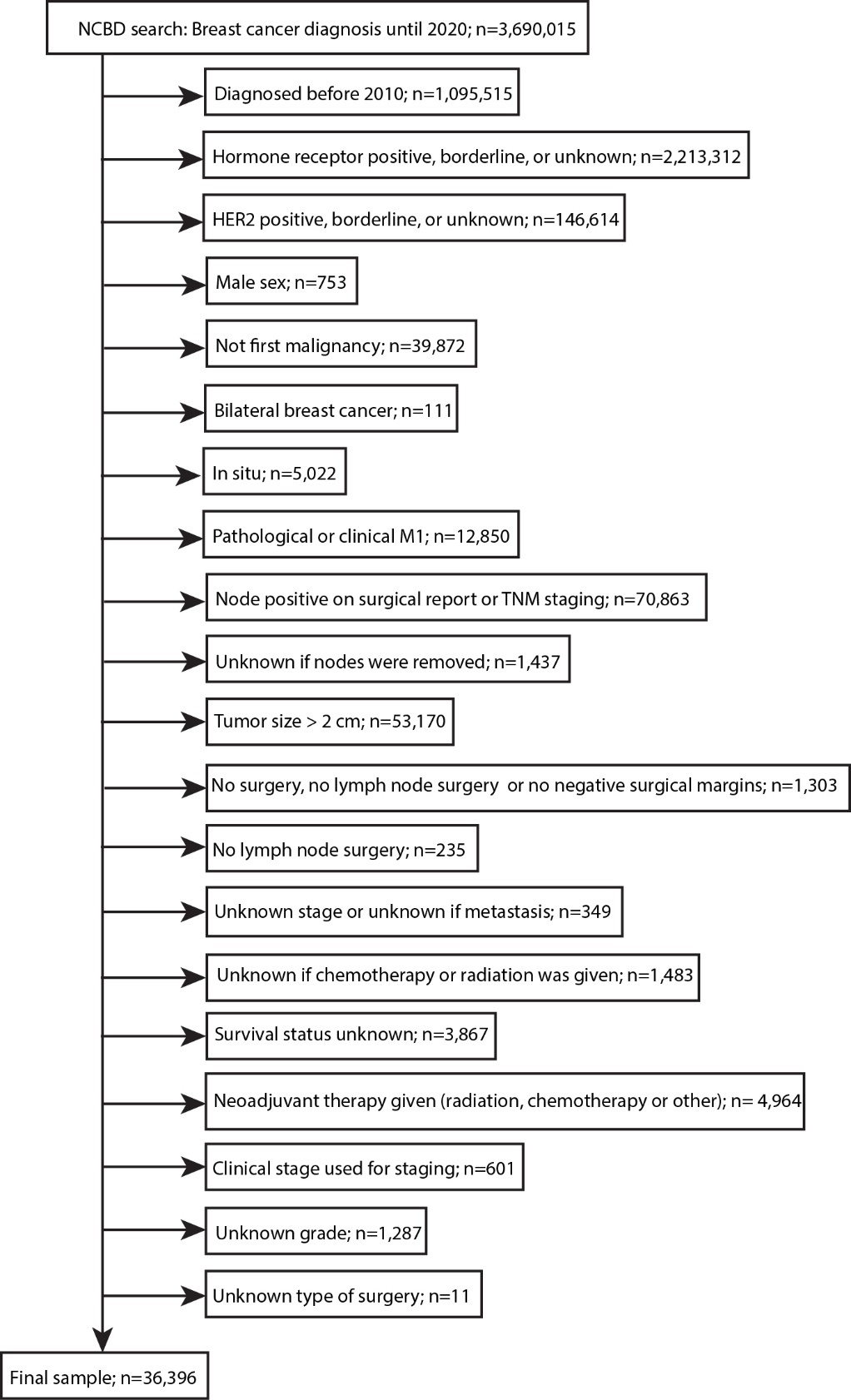


**Supplementary Figure S1.** Strobe diagram of patients included for final analysis. Abbreviations: HER2, human epidermal growth factor receptor 2; n, number; NCBD, National cancer database; TNBC, triple-negative breast cancer.

**Supplementary Figure S2.** Chemotherapy use by diagnosis year and age.


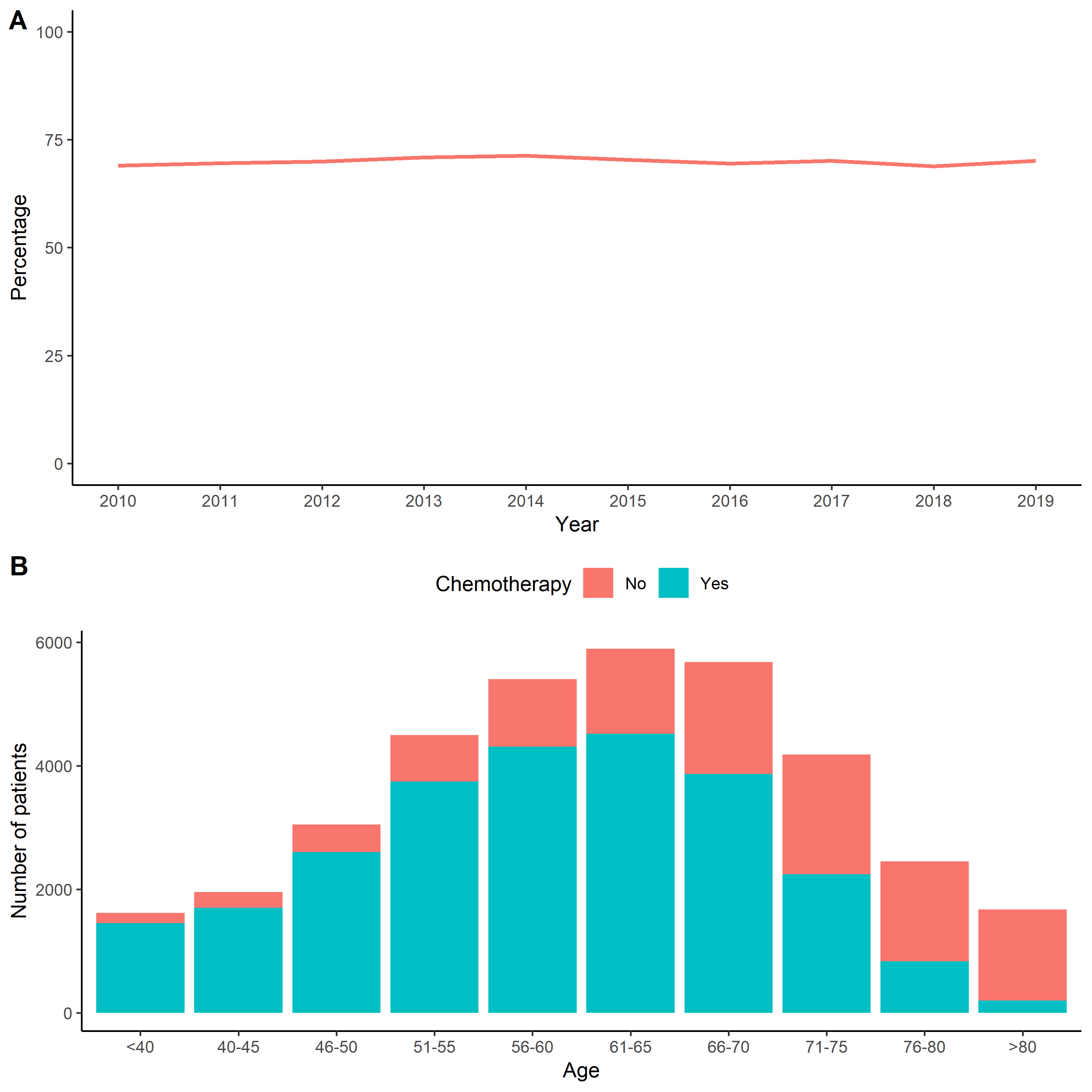


**Supplementary Figure S2.** Chemotherapy use by diagnosis year and age. A) Chemotherapy use by diagnosis year; x axis represents year of diagnosis and y axis represents percentage of patients receiving chemotherapy. B) Chemotherapy use by age; x axis represents age in years, y axis represents number of patients.

**Supplementary Figure S3.** Chemotherapy use over time stratified by tumor size


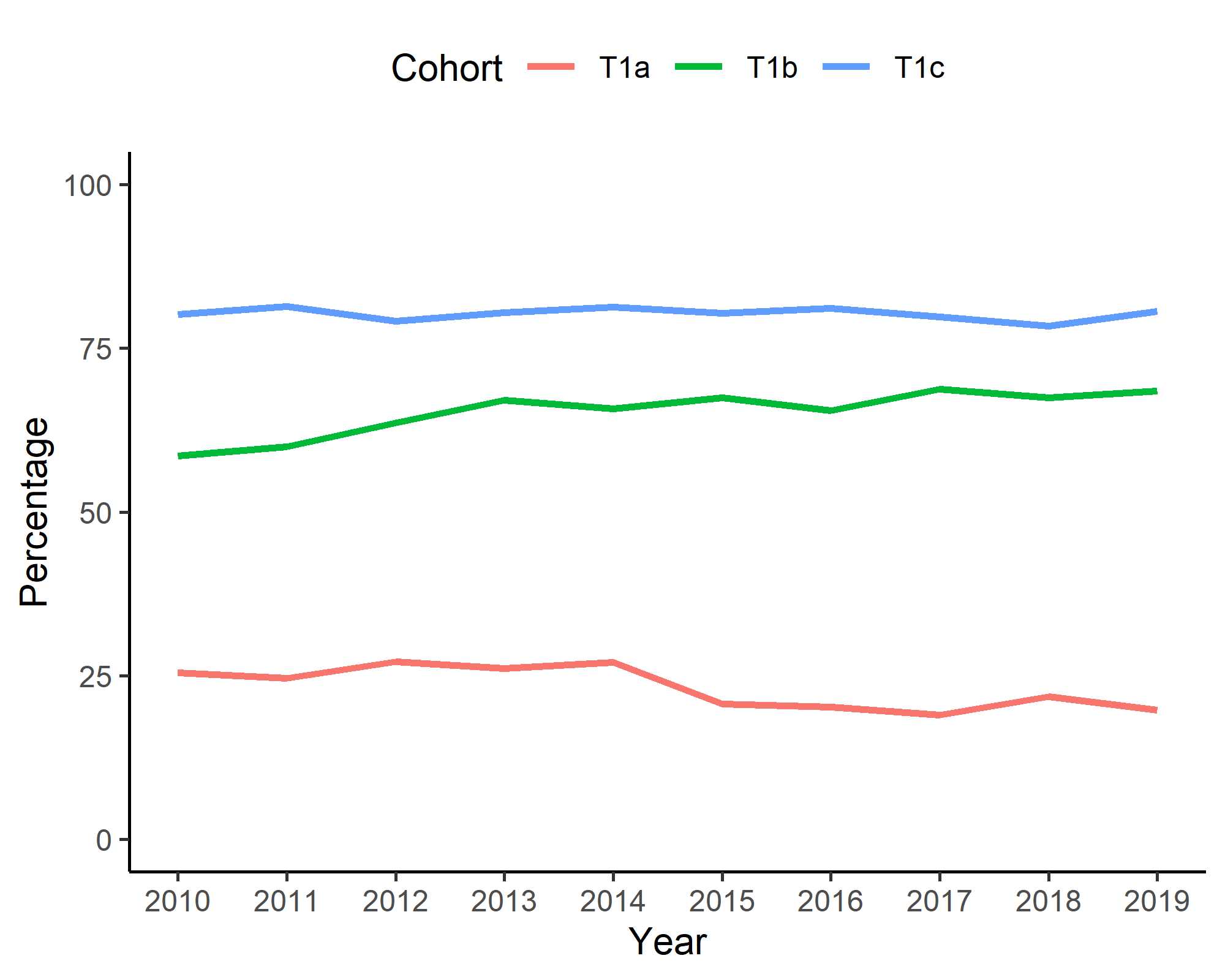


**Supplementary Figure S3.** Chemotherapy use over time stratified by tumor size. X axis represents year of diagnosis, y axis represents percentage of patients.

**Supplementary Figure S4.** Overall survival in patients with pT1N0M0 triple-negative breast cancer stratified by surgery type.


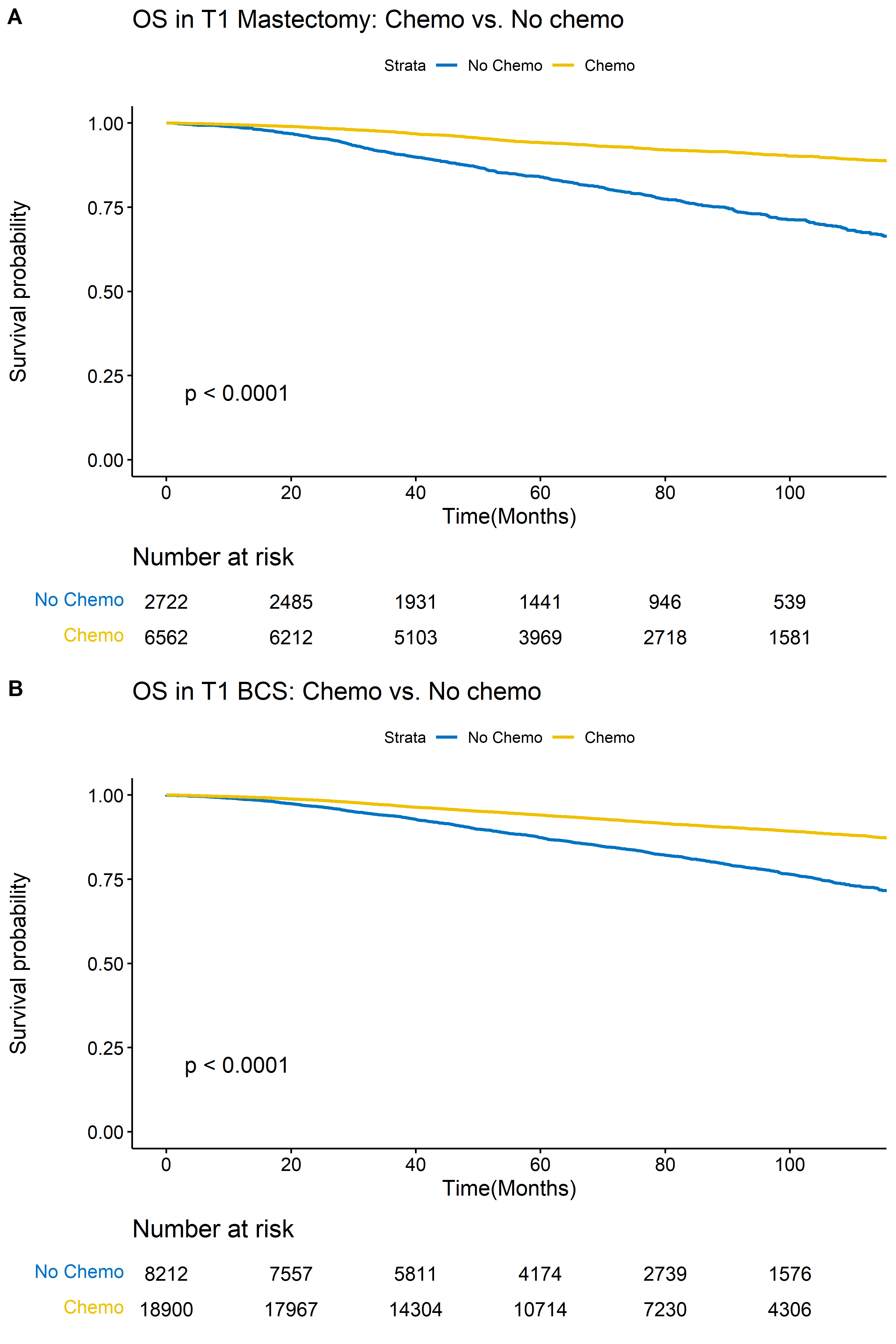


**Supplementary Figure S4.** Overall survival in patients with pT1N0M0 triple-negative breast cancer stratified by surgery type. A) Overall survival in patients with mastectomy; B) Overall survival in patients with BCS. Abbreviations: BCS, breast-conserving surgery; TNBC, triple-negative breast cancer.
